# Metabolic Reprogramming via ACOD1 depletion enhances function of human induced pluripotent stem cell-derived CAR-macrophages in solid tumors

**DOI:** 10.1038/s41467-023-41470-9

**Published:** 2023-09-18

**Authors:** Xudong Wang, Siyu Su, Yuqing Zhu, Xiaolong Cheng, Chen Cheng, Leilei Chen, Anhua Lei, Li Zhang, Yuyan Xu, Dan Ye, Yi Zhang, Wei Li, Jin Zhang

**Affiliations:** 1 Center for Stem Cell and Regenerative Medicine, Department of Basic Medical Sciences, and Bone Marrow Transplantation Center of the First Affiliated Hospital, Zhejiang University School of Medicine, Hangzhou, 310003 China; 2 Liangzhu Laboratory, Zhejiang University, Hangzhou, Zhejiang 311121 China; 3 Quanzhou First Hospital Affiliated to Fujian Medical University, Quanzhou, 362000 China; 4 Center for Stem Cell and Translational Medicine, School of Life Sciences, Anhui University, Hefei, Anhui 230601 China; 5 Center for Genetic Medicine Research, Children’s National Hospital, 111 Michigan Ave NW, Washington, DC 20010 USA; 6 Department of Genomics and Precision Medicine, George Washington University, 111 Michigan Ave NW, Washington, DC 20010 USA; 7 Shanghai Key Laboratory of Clinical Geriatric Medicine, Shanghai, Huadong Hospital, and Shanghai Key laboratory of Medical Epigenetics, International Co-laboratory of Medical Epigenetics and Metabolism (Ministry of Science and Technology), and Molecular and Cell Biology Lab, Institutes of Biomedical Sciences, Shanghai Medical College of Fudan University, Shanghai, 200032 China; 8 CellOrigin Inc, Hangzhou, 310000, China; 9 Department of General Surgery, Huashan Hospital, Fudan University, Shanghai, 200040 China; 10 Institute of Hematology, Zhejiang University, Hangzhou, 310058, China; 11 Center of Gene/Cell Engineering and Genome Medicine of Zhejiang Province, Hangzhou, 310000, China

**Keywords:** Cell delivery, Stem-cell differentiation, Monocytes and macrophages, Cancer immunotherapy

## Abstract

The pro-inflammatory state of macrophages, underpinned by their metabolic condition, is essentially affecting their capacity of combating tumor cells. Here we find, via a pooled metabolic gene knockout CRISPR screen that KEAP1 and ACOD1 are strong regulators of the pro-inflammatory state in macrophages. We show that ACOD1 knockout macrophages, generated in our induced pluripotent stem cell-derived CAR-macrophage (CAR-iMAC) platform, are strongly and persistently polarized toward the pro-inflammatory state, which manifests in increased reactive oxygen species (ROS) production, more potent phagocytosis and enhanced cytotoxic functions against cancer cells in vitro. In ovarian or pancreatic cancer mouse models, ACOD1-depleted CAR-iMACs exhibit enhanced capacity in repressing tumors, leading to increased survival. In addition, combining ACOD1-depleted CAR-iMACs with immune checkpoint inhibitors (ICI), such as anti-CD47 or anti-PD1 antibodies, result in even stronger tumor suppressing effect. Mechanistically, the depletion of ACOD1 reduces levels of the immuno-metabolite itaconate, allowing KEAP1 to prevent NRF2 from entering the nucleus to activate an anti-inflammatory program. This study thus lays down the proof of principle for targeting ACOD1 in myeloid cells for cancer immunotherapy and introduces metabolically engineered human iPSC-derived CAR-iMACs cells with enhanced polarization and anti-tumor functions in adoptive cell transfer therapies.

## Abstract (web summary)

The functional-metabolic state of macrophages fundamentally influences the tumour microenvironment, making adoptive cell therapy with pro-inflammatory macrophages an attractive anti-tumour approach. Here authors introduce pluripotent stem cell-derived CAR-macrophage that are depleted of ACOD1, an essential gene in itaconate metabolism, which reprograms them to a pro-inflammatory state enabling enhanced anti-tumour function.

## Introduction

Macrophages serve as the first line of host defense and play a key role in innate immunity. The primary function of macrophages is phagocytosis and microbial killing^1^. They also participate in a variety of physiological and pathological processes such as development, inflammation and tumorigenesis. Macrophages can be generally defined into two highly plastic states: LPS and IFN-γ-activated pro-inflammatory macrophages (M1-like macrophages) and IL-4 or IL-10 induced alternatively activated macrophages (M2-like macrophages)^2^. Recent studies revealed different metabolic pathways are closely associated with the different states. Pro-inflammatory macrophages mainly rely on glycolysis, exhibit the impaired tricarboxylic acid (TCA) cycle and express the Inducible Nitric Oxide Synthase (iNOS), whereas alternatively activated macrophages mainly rely on mitochondrial oxidative phosphorylation (OXPHOS)^3^.

Macrophages are highly plastic cells that can adapt to their surrounding environment. Pro-inflammatory M1-like macrophages play a crucial role in responding to viruses and bacteria infection and participate in anti-tumor immunity^4^, whereas M2-like macrophages can contribute to tumor progression^4^. Tumors can recruit and reprogram macrophages to become the M2-like tumor-associated macrophages (TAM). TAMs suppress endogenous cytotoxic T cells, secrete chemokines to recruit Treg cells^5^, and secrete factors such as VEGF and matrix metalloproteinase enzymes to remodel the tumor microenvironment (TME), promoting tumor angiogenesis and metastasis^6^. Thus, a primary goal of macrophage-based cancer immunotherapy is to reduce anti-inflammatory macrophages and increase pro-inflammatory macrophages.

One of the strategies targeting macrophages is to inhibit TAMs in situ in the TME. For instance, an inhibitor of the CSF-1 receptor (CSF-1R) could significantly reduce TAMs and block glioma progression in a mouse model^7^. An alternative strategy is to modify macrophages ex vivo through genetically engineered monocytes and macrophages, and the engineered macrophages can be adoptively transferred to tumor-carrying mice. A modified lentiviral vector, Vpx-LV^8^, and chimeric adenoviral vector Ad5f35^9^ were used to efficiently transduce primary monocytes and macrophages. We developed the iPSC-derived engineered CAR-macrophage (CAR-iMAC), which may become a powerful source of engineered macrophage for immunotherapy due to its ease of engineering and adequate supply. We also demonstrated antigen-dependent anti-tumor functions when challenged with antigen-expressing cancer cells in vitro and in vivo^10^. However, the first generation of CAR-iMACs was not designed to assume a pro-inflammatory state, necessitating further engineering in this direction. In this study, we used pooled CRISPR-Cas9 screens to identify the metabolic regulators of macrophage pro-inflammatory activation. Our screen revealed that the ACOD1/KEAP1/NRF2 pathway regulates cellular metabolism and pro-inflammatory activity of macrophages. Moreover, *ACOD1* depleted iMACs or CAR-iMACs are superior in comparison to the unmodified ones in cancer immunotherapies because of their enhanced in vitro and in vivo anti-tumor functions. Therefore, the present work highlights a myeloid target in cancer immunotherapy and provides reliable engineering strategies for adoptive cell transfer therapies using metabolically rewired CAR-macrophages.

## Results

### A CRISPR screen identified *KEAP1* deletion abrogated LPS and IFN-γ induced pro-inflammatory activation of macrophages

**Fig. 1:**
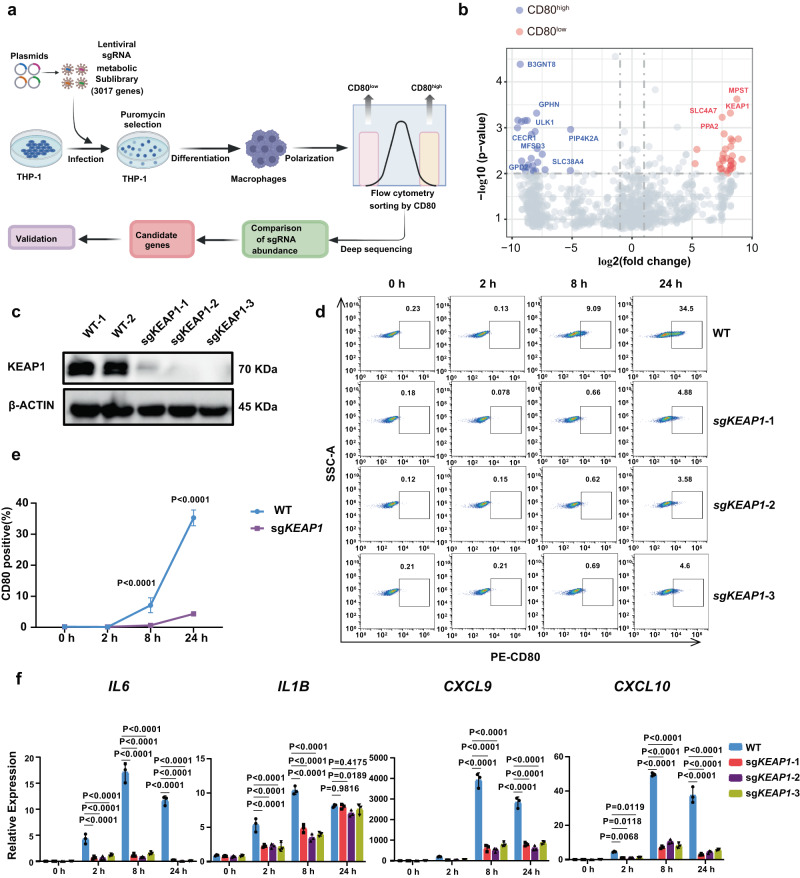
A CRISPR screen identified *KEAP1* deletion abrogated LPS and IFN-γ induced pro-inflammatory activation in macrophages. **a** A schematic diagram of the pooled CRISPR screen of metabolic genes in THP1-induced macrophages (tMAC). **b** A volcano plot displaying sgRNA-targeted genes enriched in the CD80^high^ (blue) and CD80^low^ (red) populations. **c** The protein level of KEAP1 in WT and *KEAP1*-deficient tMACs. **d**, **e** Flow cytometry plots and quantification of CD80 expression on WT and *KEAP1*-deficient tMACs after LPS and IFN-γ stimulation for 0, 2, 8, and 24 h. **e** n = 3 biologically independent samples. Statistics by two-way ANOVA test. (8 h, P < 0.0001; 24 h, P < 0.0001) **f** qRT-PCR analysis of *IL6, IL1B, CXCL9*, and *CXCL10* expression in WT and *KEAP1*-deficient tMACs after LPS and IFN-γ stimulation at different time points (n = 3 biologically independent samples). Statistics by two-way ANOVA test. **e**, **f** The data were displayed as mean ± SD. Source data are provided as a Source Data file.

To identify the possible genes influencing macrophage pro-inflammatory activation, we designed a CRISPR screen^11^ using a human metabolic sgRNA library containing metabolism-related transcription factors, small molecule transporters, and metabolic enzymes in a Cas9-expressing lentiviral vector^12^. The THP-1 cell line is a convenient system for studying human macrophages in vitro, as the THP-1 cells could be induced into macrophages by Phorbol 12-myristate 13-acetate (PMA) stimulation (Supplementary Fig. 1a) and THP-1-derived macrophages (tMAC) could be further activated towards pro-inflammatory macrophages after LPS and IFN-γ stimulation (Supplementary Fig. 1b). No significant differences in the pro-inflammatory activation capacity were found between WT and the sgRNA library virus transduced THP-1 cells (Supplementary Fig. 1c). After transduction and selection, THP-1 cells were differentiated into macrophages and stimulated with LPS and IFN-γ for 24 h. CD80^high^ and CD80^low^ populations were sorted using flow cytometry. Top ranking candidate genes enriched in the two populations were unraveled using deep sequencing (Fig. 1a). GO analysis revealed that in the CD80^high^ population, sgRNAs/genes related to NAD activity, hypoxia, and amino acid transporter were enriched, whereas in the CD80^low^ population, sgRNAs/genes related to reactive oxygen species, glycosaminoglycan biosynthesis, and glycolysis were enriched (Supplementary Fig. 1d). The screen results were also visualized with a volcano plot, which revealed that the sgRNAs targeting *KEAP1* was significantly enriched in the CD80^low^ population (Fig. 1b), and sgRNA counts of *KEAP1* were significantly higher in the CD80^low^ population (Supplementary Fig. 1e). During our screen, sg*NFKB1*s were also enriched in the CD80^low^ population (Supplementary Fig. 1f left), which is consistent with its role in promoting the inflammation program^13^. When *NFKB1* was deleted in THP-1 cells (Supplementary Fig. 1g), the expression of CD80 was significantly abrogated in the LPS and IFN-γ-induced macrophages (Supplementary Fig. 1h, i). This demonstrated that the validity of our screen in THP-1 cells was credible. We subsequently deleted *KEAP1* in THP-1 cells to validate the effect of KEAP1 on macrophage pro-inflammatory activation. We designed three sgRNAs to target the human *KEAP1* (Supplementary Fig. 2a) with good efficiency (Supplementary Fig. 2b, c), and the KEAP1 level could be reduced in THP1 cells (Fig. 1c and Supplementary Fig. 2d). To examine activation of the *KEAP1*^-/-^ tMACs, we treated tMACs with LPS and IFN-γ for 2, 8, and 24 h. The expression of CD80 is significantly abrogated in *KEAP1*^-/-^ tMACs after 8 and 24 h of stimulation (Fig. 1d, e). The expression of pro-inflammatory genes was also reduced in *KEAP1*^-/-^ tMACs, with the maximal difference between WT and *KEAP1*^-/-^ tMACs observed after 8 h of stimulation (Fig. 1f). As sg*KEAP1*−3 showed the highest efficiency (Supplementary Fig. 2c, d), sg*KEAP1*−3 transduced tMACs were used for further analysis. With RNA-seq analysis, we identified genes related to Toll-like receptor signaling pathway, phagosome, NOD-like receptor signaling pathway, and NF-κB signaling pathway was higher in WT tMACs after stimulation (Supplementary Fig. 3a), whereas genes related to Glutathione metabolism and Oxidative phosphorylation was higher in *KEAP1*^-/-^ tMACs with the same simulation (Supplementary Fig. 3b). Together, these findings indicate that *KEAP1* deletion inhibits the pro-inflammatory activation of tMACs.

We then tried to examine whether *KEAP1* had the same effects on human iMACs. We obtained an iPSC cell line with a 22 bp deletion on the *KEAP1* gene using the CRISPR-Cas9 technology (Supplementary Fig. 4a). KEAP1 protein expression was significantly decreased in the knockout cell line (Supplementary Fig. 4b). However, *KEAP1* was continuously expressed during the differentiation process from WT iPSC to iMACs (Supplementary Fig. 4c), and we could not obtain differentiated iMACs from the *KEAP1* knockout iPSC line, suggesting *KEAP1* may be an essential gene in the process of iMAC differentiation.

### *ACOD1* deletion promoted pro-inflammatory activation in tMACs

**Fig. 2:**
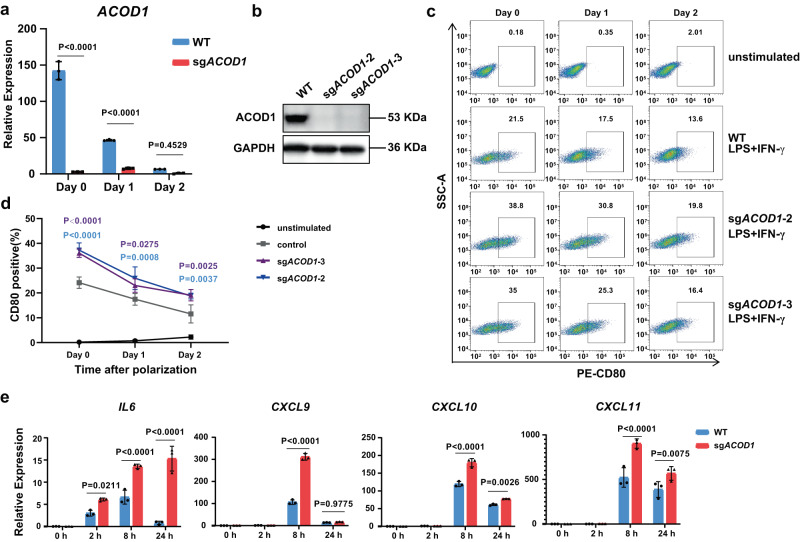
*ACOD1* deletion promoted pro-inflammatory activation in THP1-induced macrophages. **a** The relative expression of *ACOD1* in WT and sg*ACOD1* transduced THP1-induced macrophages (tMAC) with LPS and IFN-γ stimulation at the indicated time points (n = 3 biologically independent samples). Statistics by two-way ANOVA test. (Day 0, P < 0.0001; Day 1, P < 0.0001; Day 2, P = 0.4529) **b** The protein level of ACOD1 in WT and sg*ACOD1*-transduced cells after LPS and IFN-γ stimulation for 24 h. This experiment has been repeated for three times with similar results. **c**, **d** Flow cytometry plots and quantification of CD80 expression in unstimulated, WT, and sg*ACOD1* transduced tMACs with indicated treatments (**d**, n = 3 biologically independent samples). Statistics by two-way ANOVA test. The tMACs were stimulated by 50 ng/mL LPS and 50 ng/mL IFN-γ for 24 h, then withdrawn from the stimulation and further cultured for 24 h (Day 1) or 48 h (Day 2). **e** qRT-PCR for mRNA expression of pro-inflammatory genes in WT and sg*ACOD1* transduced tMACs after LPS and IFN-γ stimulation at different time points (n = 3 biologically independent samples). Statistics by two-way ANOVA test. (*IL6*: 2 h, P = 0.0211; 8 h, P < 0.0001; 24 h, P < 0.0001. *CXCL9*: 8 h, P < 0.0001; 24 h, P = 0.9755. *CXCL10*: 8 h, P < 0.0001; 24 h, P = 0.0026. *CXCL11*: 8 h, P < 0.0001; 24 h, P = 0.0075.) **a**, **d**, **e** Data was shown as mean ± SD. Source data are provided as a Source Data file.

The challenge of obtaining *KEAP1*-deleted iMACs enabled us to examine other players in the pathway. The KEAP1 protein can be modified and regulated via the alkylation of cysteine by a metabolite called itaconate^14^. Aconitate decarboxylase 1 (encoded by *ACOD1*) or Immune Responsive Gene 1 (*Irg1*) is the sole enzyme responsible for itaconate production and functions as an upstream regulator of KEAP1^15^. The alkylation of KEAP1 allows newly synthesized NRF2 to accumulate, transfer to the nucleus, and activate the transcription of anti-oxidant genes^16,17^. According to this mechanism, we speculate *ACOD1* deletion may enhance the pro-inflammatory activation of macrophages, opposite to what KEAP1 does. Our CRISPR screen in iMACs also identified sgRNAs targeting *ACOD1* enriched in the CD80^high^ population (Supplementary Fig. 5a). To further investigate the role of ACOD1 in human macrophage pro-inflammatory activation, we designed 4 sgRNAs targeting *ACOD1* (Supplementary Fig. 5b), T7 endonuclease assays revealed that sgRNA-2 and sgRNA-3 had higher cleavage activity (Supplementary Fig. 5c, d and e). We then generated *ACOD1*-deleted tMACs in which the mRNA expression was significantly lower (Fig. 2a), and the protein expression of ACOD1 was nearly blank in sg*ACOD1*−2 and sg*ACOD1*−3 tMACs (Fig. 2b). To reveal the function of ACOD1 in pro-inflammatory activation of macrophages, we found CD80 expression was higher in *ACOD1-*deleted tMACs after stimulation and the magnitude of difference was maintained after two days (Fig. 2c, d). The mRNA expression of pro-inflammatory genes showed an approximately 2-fold increase in *ACOD1-*deleted tMACs, such as *IL6* and chemokine genes *CXCL9*, *CXCL10*, and *CXCL11*, especially after 8 h of stimulation (Fig. 2e). When stimulated by LPS alone, about a 5-fold increase of CD80 expression could also be detected (Supplementary Fig. 5f, g). Collectively, these data demonstrate that *ACOD1* deletion promotes more sustainable pro-inflammatory activation of tMACs following stimulation.

### *ACOD1-*deleted human iMACs demonstrated enhanced pro-inflammatory activation

**Fig. 3:**
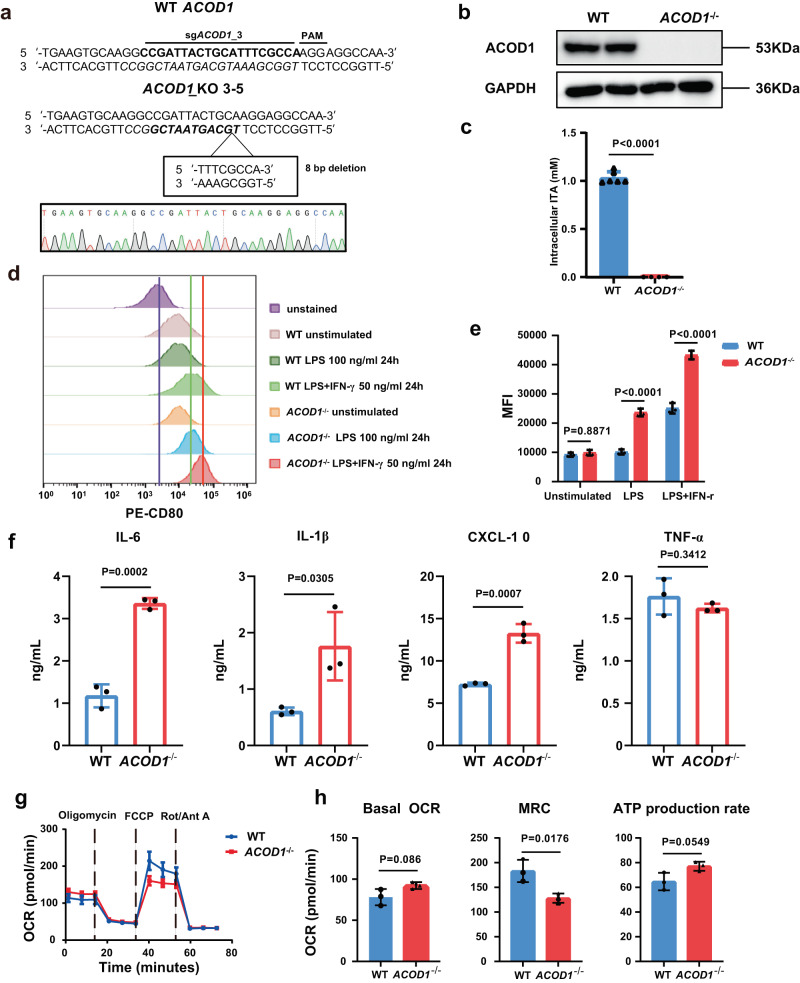
*ACOD1*-deleted human iPSC-derived macrophages demonstrated enhanced pro-inflammatory activation. **a** Comparison of the DNA sequence in the *ACOD1* knockout iPSC clone (by Sanger sequencing) with the *ACOD1* WT DNA sequence showed an 8 bp deletion in the sgRNA targeted region. **b** Western blotting for ACOD1 expression in WT and *ACOD1*^-/-^ iPSC-derived macrophages (iMAC) after LPS and IFN-γ stimulation for 24 h. This experiment has been repeated for three times with similar results. **c** Mass spectrometry quantification of the cellular itaconate (ITA) concentration in WT and *ACOD1*^-/-^ iMACs after LPS and IFN-γ stimulation for 24 h (WT, n = 6 biologically independent samples; *ACOD1*^-/-^, n = 4 biologically independent samples). Statistics by unpaired t test. (P < 0.0001) **d**, **e** CD80 expression on WT and *ACOD1*^-/-^ iMACs and quantification was determined by flow cytometry under different treatments, including 100 ng/mL LPS or 50 ng/mL LPS plus 50 ng/mL IFN-γ stimulation for 24 h (e, n = 3 biologically independent samples). Statistics by two-way ANOVA test. (unstimulated, P = 0.8871; LPS, P < 0.0001; LPS + IFN-γ, P < 0.0001) **f** The levels of the indicated cytokines/chemokines in the medium of iMAC culturing were determined 24 h post IFN-γ and LPS challenge (n = 3 biologically independent samples). Statistics by unpaired t test. **g** Seahorse extracellular metabolic flux analysis of oxygen consumption rates (OCR). LPS and IFN-γ stimulated WT or *ACOD1*^-/-^ iMACs were sequentially treated with oligomycin (1.5 μM), fluorcarbonylcyanide phenylhydrazone (FCCP; 2 μM), and rotenone and antimycin A (0.5 μM each) (n = 3). **h** Basal OCR, maximal respiration capacity (MRC), and ATP production rate were calculated with Wave 2.4.0. (n = 3 biologically independent samples). Statistics by unpaired t test. **c**, **e**, **f**, **g** and **h** Data was shown as mean ± SD. Source data are provided as a Source Data file.

To investigate whether *ACOD1* deletion contributes to pro-inflammatory activation in human iMACs, we knocked out *ACOD1* in human iPSC using the CRISPR/Cas9 technology with sgRNA-3. A cell line with an 8 bp deletion on the fourth exon of the *ACOD1* gene was established (Fig. 3a). Differentiation from this engineered iPSCs to iMACs was successful, and the purity of iMACs reached to 96% on day 29 (Supplementary Fig. 6a, b). The deficiency of ACOD1 in mRNA (Supplementary Fig. 6c) and protein expression (Fig. 3b) was confirmed. As expected, the intracellular concentration of itaconate was also significantly lower in *ACOD1* deficient iMACs (Fig. 3c). After 24 h of stimulation with LPS or LPS plus IFN-γ, the expression of CD80 was significantly higher in *ACOD1*^-/-^ iMACs (Fig. 3d, e). We further measured the mRNA expression of other pro-inflammatory genes to confirm this result. In line with elevated CD80 expression, pro-inflammatory genes *IL6, IL1B, IL1A, IL23A* and *CXCL10* were also significantly higher in *ACOD1*^-/-^ iMACs (Supplementary Fig. 6d). We also validated the changes at the protein level with ELISA. Compared with WT iMACs, *ACOD1*^-/-^ iMACs had increased levels of pro-inflammatory cytokines and chemokines such as IL-6, IL-1β and CXCL-10 in the supernatant upon LPS and IFN-γ stimulation (Fig. 3f). To extend the finding that *ACOD1* restricted the iMAC pro-inflammatory state and the associated metabolic program, we measured real-time changes in cellular oxygen consumption rate (OCR) in WT and *ACOD1*^-/-^ iMACs. *ACOD1* deletion led to a decreased OCR (Fig. 3g), including decreased maximal respiration capacity (MRC) (Fig. 3h), suggesting a decrease in mitochondrial function typically associated with the M2-like state in the absence of *ACOD1*. Together, these results demonstrate that *ACOD1* deletion promotes pro-inflammatory activation of iMACs, and decreases mitochondrial function upon pro-inflammatory stimulation.

### *ACOD1*^-/-^ iMACs demonstrated a stronger phagocytosis and anti-cancer cell function

**Fig. 4:**
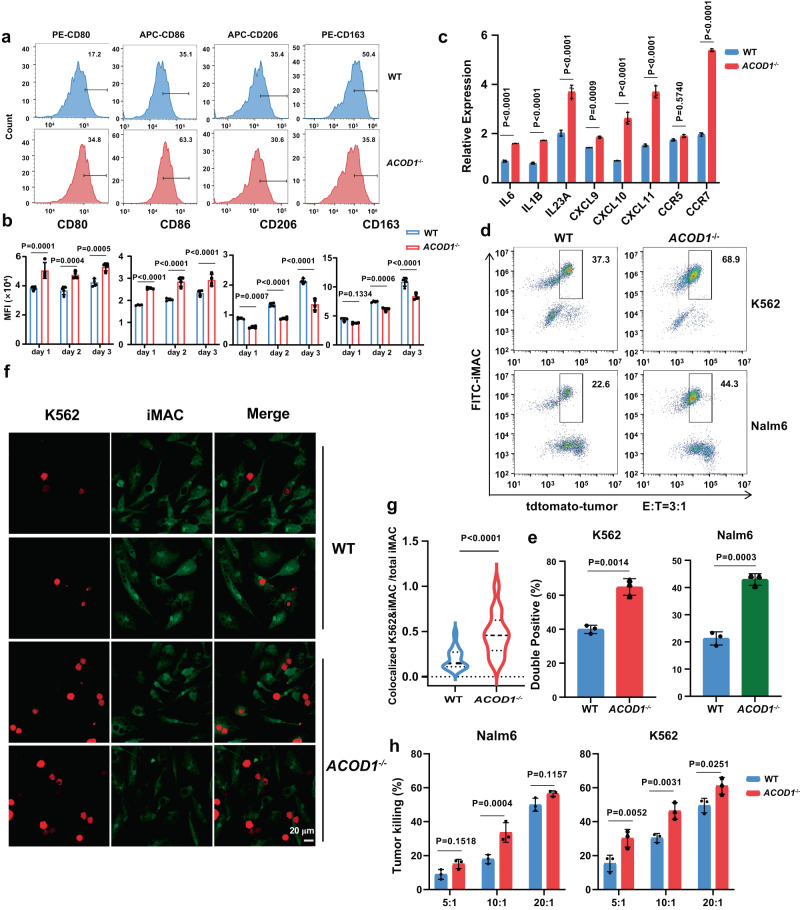
*ACOD1*^-/-^ iMACs had stronger phagocytosis and anti-cancer cell function. **a** CD80, CD86, CD163, and CD206 expression in WT or *ACOD1*^-/-^ iMACs after co-cultured with Nalm6 (E:T = 5:1) for 24 h were measured by flow cytometry and displayed as histograms. **b** Quantification of mean fluorescence intensity (MFI) measured by flow cytometry after co-cultured with Nalm6 (E:T = 5:1) for 24 h (day 1), 48 h (day 2), or 72 h (day 3) (n = 4 biologically independent samples). Statistics by two-way ANOVA test. (CD80: day 1, P = 0.0001; day 2, P = 0.0004; day 3, P = 0.0005. CD86: day 1, P < 0.0001; day 2, P < 0.0001; day 3, P < 0.0001. CD206: day 1, P = 0.0007; day 2, P < 0.0001; day 3, P < 0.0001. CD163: day 1, P = 0.1334; day 2, P = 0.0006; day 3, P < 0.0001.) **c** qRT-PCR for mRNA expression of pro-inflammatory genes in WT and *ACOD1*^-/-^ iMACs after co-cultured with Nalm6 (E:T = 5:1) for 24 h (n = 3 biologically independent samples). Statistics by two-way ANOVA test. (*IL6, IL1B, IL23A, CXCL10, CXCL11* and *CCR7*, P < 0.0001. *CXCL9*, P = 0.0009.) **d**, **e** Representative flow cytometry plots and quantification of double positive iMACs after WT and *ACOD1*^-/-^ iMACs were co-cultured with Nalm6 and K562 cells (E:T = 3:1) for 24 h (e, n = 3 biologically independent samples). Statistics by unpaired t test. **f**, **g** Representative confocal images and quantification of K562 cells phagocytosed by WT or *ACOD1*^-/-^ iMACs after co-cultured for 24 h (g, WT, n = 27 views from 3 biologically independent samples; *ACOD1*^-/-^, n = 14 views from 3 biologically independent samples). Statistics by unpaired t test. (P < 0.0001) Representative confocal images were obtained using the Olympus FV3000 microscope and ImageJ software. The number of colocalized K562&iMAC and total iMAC in one view was used to calculate the ratio. **h** Luciferase assays showing iMAC cytotoxicity against cancer cells when co-cultured wit**h** Nalm6 or K562 cells for 24 h (E:T = 5:1, 10:1, or 20:1) (n = 3 biologically independent samples). Statistics by two-way ANOVA test. The luciferase gene has been introduced by lentivirus to tumor cells and expressed in tumor cells, so that tumor cell viability can be measured by D-luciferin sodium salt in a luciferase assay. **b**, **c**, **g**, **e**, **h** Data was shown as mean ± SD. Source data are provided as a Source Data file.

To further investigate the role of ACOD1 in iMACs in the presence of tumor cells, Nalm6 or K562 cells were used to co-culture with WT iMACs or *ACOD1*^-/-^ iMACs. We found that, after co-culturing with Nalm6 cells for 24 h at an effector: target (E:T) ratio of 5:1 or 3:1, the expression levels of M1-like markers CD80 and CD86 were higher in *ACOD1*^-/-^ iMACs, whereas M2-like markers CD163 and CD206 were lower (Fig. 4a and Supplementary Fig. 7a). Co-culturing with K562 cells had the similar results (Supplementary Fig. 7b, c). Importantly, a long-term co-culture assay revealed that the expression of M1-like markers remained elevated, whereas M2-like markers remained lower in *ACOD1*^-/-^ iMACs in three days (Fig. 4b), indicating that *ACOD1* deletion could contribute to a long-term maintenance of higher pro-inflammatory activation and resistance to conversion toward the anti-inflammatory state in the presence of Nalm6 tumor cells. In addition, mRNA expression of other M1-like marker genes was also significantly higher in *ACOD1*^-/-^ iMACs co-cultured with Nalm6 cells (Fig. 4c and Supplementary Fig. 7d), and their expression was also maintained higher over long term co-culturing (Supplementary Fig. 7e). Next, flow cytometry results support the stronger phagocytosis function of *ACOD1*^-/-^ iMACs against tumor cells (Fig. 4d, e). The isotype control and gating strategy of the phagocytosis assay was shown in Supplementary Fig. 7f–h. Confocal imaging analysis also showed that *ACOD1*^-/-^ iMACs co-cultured with K562 cells for 24 h had a stronger phagocytosis function (Fig. 4f, g). Finally, the luciferase assay showed *ACOD*1^-/-^ iMACs had higher cytolytic activity against tumor cells (Fig. 4h). Taken together, the above data demonstrate *ACOD1* deletion promotes a stronger anti-tumor function of iMACs upon tumor cell stimulation.

### *ACOD1* deletion decreased the expression of the nuclear NRF2 protein and its activity in iMACs

**Fig. 5:**
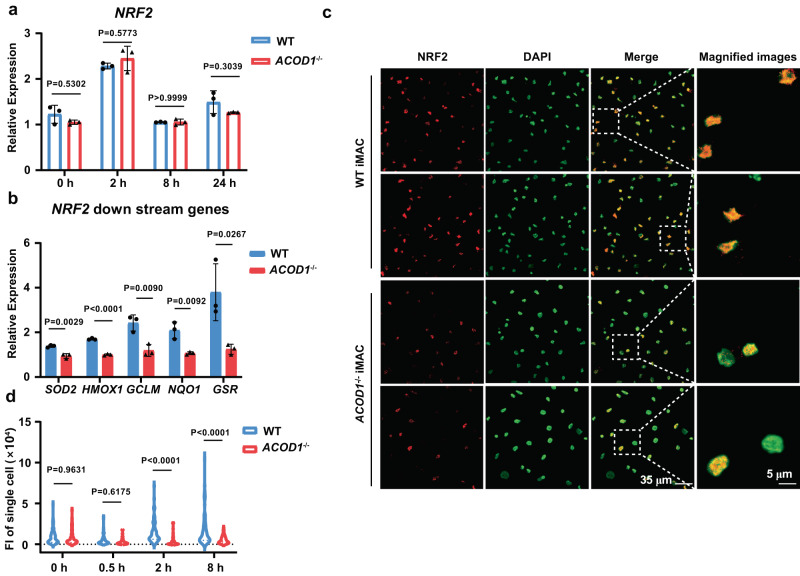
*ACOD1* deletion decreased nucleolar NRF2 protein expression and its activity in iMACs. **a** qRT-PCR for mRNA expression of *NRF2* in WT and *ACOD1*^-/-^ iMACs after LPS and IFN-γ stimulation for 2, 8, or 24 h (n = 3 biologically independent samples). Statistics by two-way ANOVA test. **b** qRT-PCR for mRNA expression of *NRF2* downstream genes in WT and *ACOD1*^-/-^ iMACs after LPS and IFN-γ stimulation for 24 h (n = 3 biologically independent samples). Statistics by two-way ANOVA test. (*SOD2*, P = 0.0029; *HMOX1*, P < 0.0001; *GCLM*, P = 0.009; *NQO1*, P = 0.0092; *GSR*, P = 0.0267) **c**, **d** Representative confocal images and quantification of the NRF2 protein in WT and *ACOD1*^-/-^ iMACs after LPS and IFN-γ stimulation for 2 h, fluorescence intensity (FI) of single cells was counted (d, n = 60 cells from 3 biologically independent samples each group). Statistics by two-way ANOVA test. (2 h, P < 0.0001; 8 h, P < 0.0001) This experiment has been repeated for three times with similar results. Representative confocal images were obtained using the Olympus FV3000 microscope. **a**, **b**, **d** Data was shown as mean ± SD. Source data are provided as a Source Data file.

It was demonstrated that itaconate was a crucial anti-inflammatory metabolite that acts via NRF2^14^. To understand the molecular mechanisms of *ACOD1* depletion in our iMAC system, we examined NRF2 and its downstream genes in iMACs. We found mRNA expression of *NRF2* had no significant difference in WT and *ACOD1*^-/-^ iMACs (Fig. 5a). However, the expression of *NRF2* downstream genes decreased significantly in *ACOD1*^-/-^ iMACs, such as *SOD2, HMOX1, GCLM, NQO1*, and *GSR* (Fig. 5b). Confocal imaging showed that the total NRF2 protein level in the nucleus decreased significantly in *ACOD1*^-/-^ iMACs, especially after LPS and IFN-γ stimulation for 2 and 8 h (Fig. 5c, d and Supplementary Fig. 8a–c). One of the NRF2 targets is TNFAIP3 (A20) which is a negative regulator of the NF-κB pathway and macrophage activation^18^. We measured *A20* expression in iMACs and found that it decreased in *ACOD1*^-/-^ iMACs (Supplementary Fig. 8d), which is likely to mediate increased NF-κB activity. To further validate the functional effect of NRF2 on macrophage activation, we designed three gRNAs targeting *NRF2* (Supplementary Fig. 9a), which all successfully lowered the mRNA expression of *NRF2* (Supplementary Fig. 9b). Depletion of *NRF2* recapitulated *ACOD1* deletion in that CD80 expression was higher in sg*NRF2*s transduced tMACs compared to WT controls after LPS and IFN-γ stimulation for 24 h (Supplementary Fig. 9c), and consistently mRNA expression of pro-inflammatory genes was also higher (Supplementary Fig. 9d). Together, these results demonstrate that *ACOD1* deletion decreased NRF2 activity to allow pro-inflammatory activation of iMACs.

### *ACOD1* deletion promoted anti-cancer cell activity against solid tumors of CAR-iMACs in vitro and in vivo

**Fig. 6:**
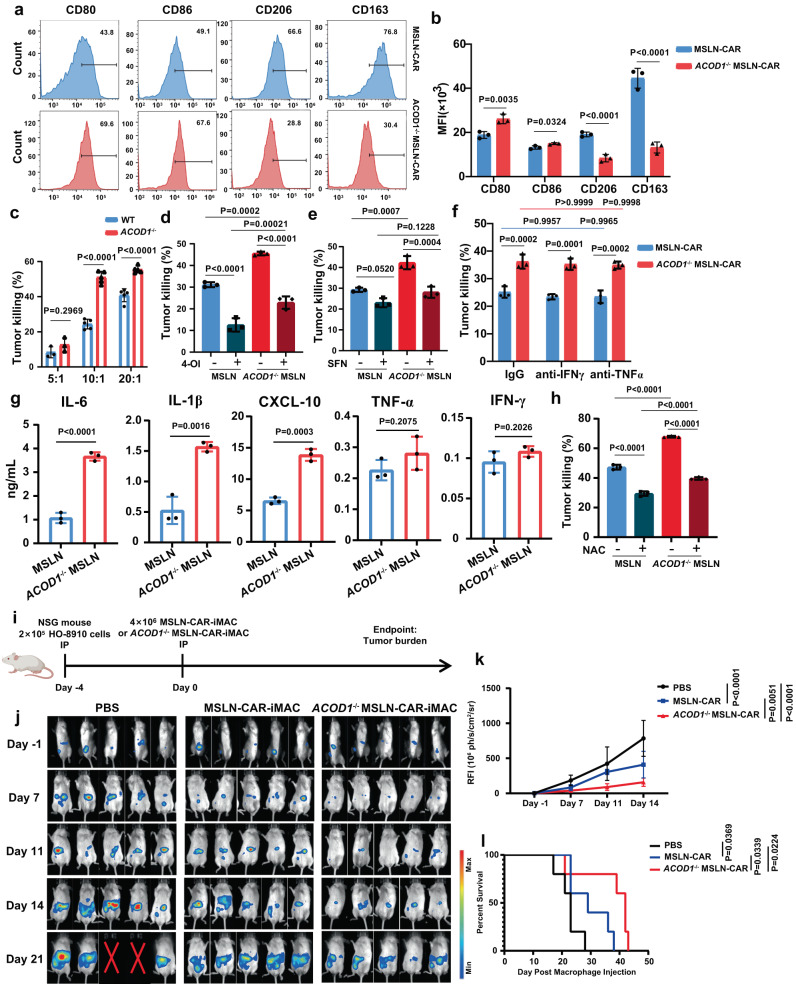
*ACOD1* deletion promoted anti-cancer cell activity of iMACs against solid tumors in vitro and in vivo. **a**, **b** The expression and quantification of CD80, CD86, CD163, and CD206 in MSLN-CAR-iMACs or *ACOD1*^-/-^ MSLN-CAR-iMACs after co-cultured with HO-8910 cells (E:T = 5:1) for 24 h were measured by flow cytometry and displayed as histograms (b, n = 3 biologically independent samples). statistics by two-way ANOVA test. (CD80, P = 0.0035; CD86, P = 0.0324; CD206, P < 0.0001; CD163, P < 0.0001) **c** Luciferase assays for CAR-iMAC cytotoxicity activity against cancer cells when co-cultured with HO-8910 cells for 24 h (E:T = 5:1, 10:1, or 20:1) (5:1, n = 3; 10:1, n = 5; 20:1, n = 5 biologically independent samples). statistics by two-way ANOVA test. (5:1, P = 0.2969; 10:1, P < 0.0001; 20:1, P < 0.0001) **d** Luciferase assays for CAR-iMAC cytotoxicity activity against cancer cells with or without 4-Octyl Itaconate (4-OI) addition when co-cultured with HO-8910 cells for 24 h (n = 3 biologically independent samples) (E:T = 10:1). Statistics by one-way ANOVA test. (MSLN(-) vs MSLN(+), P < 0.0001; *ACOD1*^-/-^ MSLN(-) vs *ACOD1*^-/-^ MSLN(+), P < 0.0001; MSLN(-) vs *ACOD1*^-/-^ MSLN(-), P = 0.0002; MSLN(+) vs *ACOD1*^-/-^ MSLN(+), P = 0.00021) iMACs were pre-treated with 4-OI (250 μM) or DMSO control for 3 h before challenge with LPS plus IFN-γ (50 ng/mL each) for 24 h. **e** Luciferase assays for MSLN-CAR-iMAC cytotoxicity activity against cancer cells with or without sulforaphane (SFN) (10 μM) when co-cultured with HO-8910 cells for 24 h (E:T = 10:1) (n = 3 biologically independent samples). Statistics by one-way ANOVA test. **f** Luciferase assays for the cytotoxicity activity of the co-culture supernatant with IgG control, neutralizing antibody (10 μg/mL) of IFN-γ or TNF-α (n = 3 biologically independent samples). Statistics by two-way ANOVA test. The supernatant was collected after iMACs were co-cultured with HO-8910 cells for 24 h (E:T = 10:1). **g** The levels of the indicated cytokines/chemokines in the medium of iMAC-HO-8910 co-culture system were determined 24 h post IFN-γ and LPS challenge (n = 3 biologically independent samples). Statistics by unpaired t test. (IL-6, P < 0.0001; IL-1β, P = 0.0016; CXCL-10, P = 0.0003; TNF-α, P = 0.2075; IFN-γ, P = 0.2026) **h** Luciferase assays for MSLN-CAR-iMAC cytotoxicity activity against cancer cells with or without N-Acetyl-L-cysteine (NAC) (2.5 mM) when co-cultured with HO-8910 cells for 48 h (E:T = 10:1) (n = 3 biologically independent samples). Statistics by one-way ANOVA test. (MSLN(-) vs MSLN(+), P < 0.0001; *ACOD1*^-/-^ MSLN(-) vs *ACOD1*^-/-^ MSLN(+), P < 0.0001; MSLN(-) vs *ACOD1*^-/-^ MSLN(-),P < 0.0001; MSLN(+) vs *ACOD1*^-/-^ MSLN(+), P < 0.0001) (**b-h**) Data was shown as mean ± SD. **i** A diagram of the in vivo treatment scheme. **j** In Vivo Imaging system (IVIS) images showing progression of tumor in the above conditions (n = 5 per group). **k** Tumor burden on day −1, 7, 11, and 14 was quantified and displayed as mean ± SD. (n = 5 per group) statistics by two-way ANOVA test. (PBS vs MSLN-CAR, P < 0.0001; PBS vs *ACOD1*^-/-^ MSLN-CAR, P < 0.0001; MSLN-CAR vs *ACOD1*^-/-^ MSLN-CAR, P = 0.0051) **l** The Kaplan-Meier curve demonstrating survival of the mice. Statistics by two-tailed log-rank test. Source data are provided as a Source Data file.

Adoptive cell therapy with genetically modified immune cells has been established as a promising approach for cancer treatment. However, applications to solid tumors have proven challenging. To improve the anti-solid tumor functions of iMACs, we used our previously established CAR-iMAC system, in which we stably expressed the first generation of anti-mesothelin (MSLN) CAR with CD3ζ as the intracellular domain in human iPSCs and differentiated them to produce MSLN-CAR-iMACs to kill mesothelin-expressing ovarian tumors both in vitro and in vivo^10^. We then performed a detailed comparison of MSLN-CAR-iMACs and *ACOD1*^-/-^ MSLN-CAR-iMACs. *ACOD1*^-/-^ MSLN-CAR-iMACs expressed more pro-inflammatory marker proteins (CD80 and CD86) after being co-cultured with HO-8910 ovarian cancer cells for 24 h (Fig. 6a, b). Lower anti-inflammatory marker proteins (CD163 and CD206) were consistently detected in *ACOD1*^-/-^ MSLN-CAR-iMACs (Fig. 6a, b). In vitro tumor cell killing assay revealed that *ACOD1*^-/-^ MSLN-CAR-iMACs significantly increased anti-tumor activity (Fig. 6c), which could be dampened by supplementing a cell permeable 4-Octyl Itaconate (4-OI)^19^ (Fig. 6d). To further dissect the downstream signaling related to NRF2, a chemical NRF2 activator sulforaphane (SFN)^20^ was added to the co-culture system, which abrogated the enhanced capacity in *ACOD1*^-/-^ MSLN-CAR-iMACs (Fig. 6e). To examine the cytolytic mechanisms in addition to phagocytosis, we collected the supernatant from the co-culture of tumor cells and CAR-iMACs, and then added it to another well of tumor cells. To our surprise, the supernatant alone had tumor killing function (Fig. 6f), which was not abrogated by neutralizing antibodies of TNF-α and IFN-γ (Fig. 6f), two cytolytic cytokines. Interestingly, compared with MSLN-CAR-iMACs, *ACOD1*^-/-^ MSLN-CAR-iMACs had increased inflammatory cytokines such as IL-6, IL-1β and CXCL-10, but not TNF-α and IFN-γ when co-cultured with HO-8910 cells for 24 h (Fig. 6g). Then we explored other potential cytolytic factors in the medium. It has been reported that reactive oxygen species (ROS) is produced upon LPS stimulation and through TLR^21^, and it can be dampened by supplementing itaconate in macrophages^14^. Compared to MSLN-CAR-iMACs, ROS production was also elevated in *ACOD1*^-/-^ MSLN-CAR-iMACs (Supplementary Fig. 10a, b). To examine the function of ROS in tumor killing ability of *ACOD1*^-/-^ MSLN-CAR-iMACs, we added the anti-oxidant reagent N-Acetyl-L-cysteine (NAC) to eliminate ROS in the tumor-iMAC co-culture system. The tumor killing capacity of CAR-iMACs was significantly blocked by NAC (Fig. 6h). This result demonstrated that ROS contributed to the enhanced tumor killing ability of *ACOD1*^-/-^ MSLN-CAR-iMACs.

To evaluate the anti-tumor activity of *ACOD1*^-/-^ MSLN-CAR-iMACs in vivo, we used two different xenograft solid tumor models with the NOD/SCID/IL2rγnull (NSG) mice. In the first model, the mice were inoculated intraperitoneally (IP) with luciferase-expressing HO-8910 cells. After four days, the mice received a single IP injection of iMACs (Fig. 6i), and were monitored by bioluminescent imaging (BLI) afterwards (Fig. 6j). Compared with the untreated or the MSLN-CAR-iMACs treated mice, treatment with *ACOD1*^-/-^ MSLN-CAR-iMACs led to significant inhibition of tumor growth (Fig. 6k). This improved anti-tumor activity also led to markedly improved survival time (Fig. 6l). Then we examined the pro-inflammatory activity of iMACs in vivo. iMACs were injected intratumorally, and the pro-inflammatory markers were significantly elevated in *ACOD1*^-/-^ MSLN-CAR-iMACs compared with unmodified MSLN-CAR-iMACs after injection for 7 days (Supplementary Fig. 11a, b) or 14 days (Supplementary Fig. 11c, d). These results indicated that *ACOD1*^-/-^ CAR-iMACs could keep an enhanced pro-inflammatory activity in vivo for at least 14 days.

Consistent results were obtained in another setting of pancreatic cancer. The M1 markers were significantly elevated in *ACOD1*^-/-^ MSLN-CAR-iMACs compared with unmodified MSLN-CAR-iMACs after co-cultured with AsPC-1 pancreatic cancer cells for 24 h (Supplementary Fig. 12a). In vitro tumor killing capacity was enhanced in *ACOD1*^-/-^ MSLN-CAR-iMACs (Supplementary Fig. 12b) which could be reversed by supplementing 4-OI (Supplementary Fig. 12c). The expression of pro-inflammatory genes was also elevated in *ACOD1*^-/-^ MSLN-CAR-iMACs (Supplementary Fig. 12d). In line with the in vitro results, in vivo assay using a pancreatic tumor mouse model with IP injected AsPC-1 cells also demonstrated the stronger anti-tumor activity of *ACOD1*^-/-^ MSLN-CAR-iMACs (Supplementary Fig. 12e–h).

### *ACOD1* deletion promoted the anti-tumor activity of MSLN-CAR-iMACs in combination with immune check point inhibitors in vivo

Tumor cells evade normal immune system via transmitting inhibitory signals to myeloid cells^22^ and lymphocytes^23^. Immune checkpoint is one of the mechanisms that regulate cancer immune escape. For instance, CD47 is expressed on many cancer cells, and binding of CD47 to signal-regulatory protein α (SIRPα) on macrophages results in inhibition of macrophage phagocytic activity^24^. Programmed cell death protein 1 (PD-1) is an immune checkpoint receptor mainly upregulated on activated T cells for the induction of immune tolerance. It’s well known that PD-1-PD-L1 blockade could activate T cells^25^. PD-1 is also expressed on TAMs, and its expression is negatively correlated with phagocytic potency of macrophages^26^. We hypothesized that the combination of CAR-iMACs with immune check point inhibitors (ICI) may enhance the anti-tumor activity. So we assessed two combination immunotherapy strategies using MSLN-CAR-iMACs with the anti-CD47 antibody and the anti-PD-1 antibody, respectively.

**Fig. 7:**
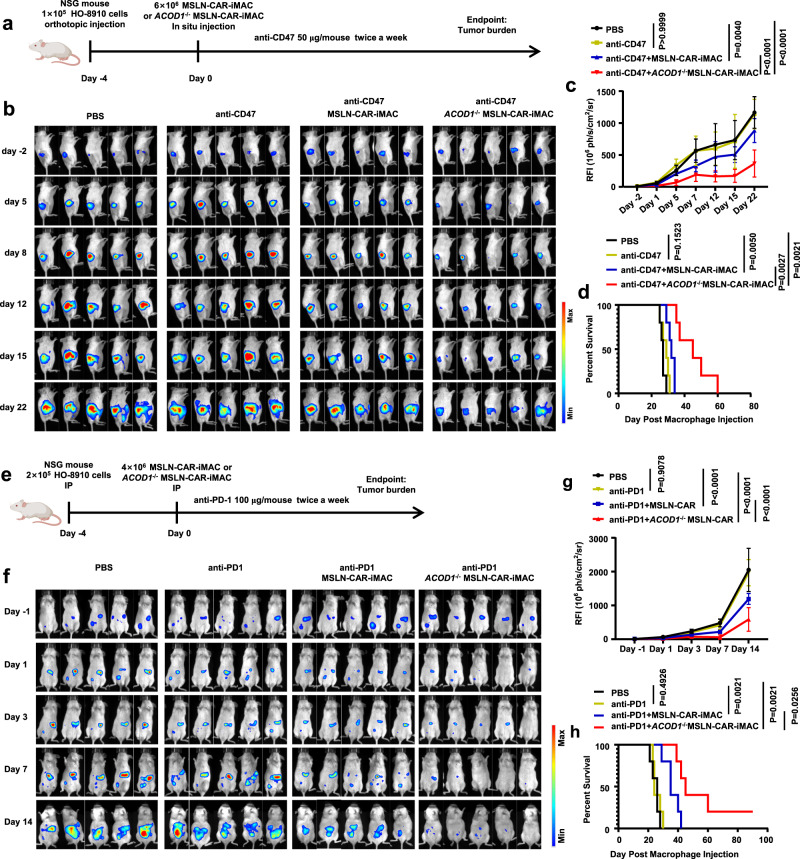
*ACOD1* deletion promoted the anti-ovarian cancer activity of iMACs combining with ICIs in vivo. **a** A schematic of the in vivo study using HO-8910 cells for a mouse ovarian orthotopic injection model treated with MSLN-CAR-iMACs and *ACOD1*^-/-^ MSLN-CAR-iMACs, and combined with an anti-CD47 antibody. **b** Tumor burden was determined by bioluminescent imaging (BLI). Images of representative time points were shown (n = 5 per group). **c** Quantification of tumor burden of representative time points was displayed as mean ± SD. Statistics by two-way ANOVA test. (PBS vs anti-CD47, P > 0.9999; PBS vs anti-CD47 + MSLN-CAR-iMAC, P = 0.004; PBS vs anti-CD47 + *ACOD1*^-/-^ MSLN-CAR-iMAC, P < 0.0001; anti-CD47 + MSLN-CAR-iMAC vs anti-CD47 + *ACOD1*^-/-^ MSLN-CAR-iMAC, P < 0.0001) **d** The Kaplan-Meier curve demonstrating survival of the mice. Statistics by two-tailed log-rank test. **e** A schematic of the in vivo study using HO-8910 cells for a mouse intraperitoneal (IP) injection model treated with MSLN-CAR-iMACs and *ACOD1*^-/-^ MSLN-CAR-iMACs, and combined with an anti-PD1 antibody. **f** Tumor burden was determined by BLI. Images of representative time points were shown (n = 5 per group). **g** Quantification of tumor burden of representative time points was displayed as mean ± SD. Statistics by two-way ANOVA test. (PBS vs anti-PD1, P = 0.9078; PBS vs anti-PD1 + MSLN-CAR, P < 0.0001; PBS vs anti-PD1 + *ACOD1*^-/-^ MSLN-CAR, P < 0.0001; anti-PD1 + MSLN-CAR vs anti-PD1 + *ACOD1*^-/-^ MSLN-CAR, P < 0.0001) **h** The Kaplan-Meier curve demonstrating survival of the mice. Statistics by two-tailed log-rank test. Source data are provided as a Source Data file.

In the first xenograft model, HO-8910 cells were inoculated through orthotopic injection at the ovary of the mice. After four days, the mice received a single in situ intratumoral injection of iMACs. At the same time, the mice received IP injections of a low dose anti-CD47 antibody, and it was kept twice a week to further enhance the function of CAR-iMACs by blocking the “don’t eat me” signal (Fig. 7a). The tumor growth was monitored by BLI (Fig. 7b). Compared with untreated tumor-bearing mice, the low-dose anti-CD47 antibody treatment alone could not inhibit tumor growth. The combination of the low-dose anti-CD47 antibody with the MSLN-CAR-iMACs could inhibit tumor growth to some extent. Importantly, the combination of low-dose anti-CD47 antibody with *ACOD1*^-/-^ MSLN-iMACs had the most superior tumor suppression effect (Fig. 7c). This improved anti-tumor activity led to markedly improved survival time compared with all other conditions (Fig. 7d). In the second xenograft model with the anti-PD-1 antibody, the mice were inoculated IP with luciferase-expressing HO-8910 cells. After four days, the mice received a single IP injection of iMACs. Meanwhile, the mice received IP injections of a low dose of the anti-PD-1 antibody, and subsequently the antibody was used twice a week to further block the PD-1-PD-L1 axis (Fig. 7e). The mice were monitored by BLI afterwards (Fig. 7f). Compared with other groups, the combination of the low-dose anti-PD1 antibody with *ACOD1*^-/-^ MSLN-CAR-iMACs had the most superior tumor suppression effect (Fig. 7g), and markedly lengthened the survival time (Fig. 7h). Together, these data strongly demonstrated that *ACOD1*-deleted CAR-iMACs combined with ICIs had the most superior anti-tumor activity.

## Discussion

The ACOD1/KEAP1/NRF2 axis plays a crucial role in maintaining redox balance and macrophage polarization in mouse and human macrophages^14^. In the mouse sepsis syndrome model, *Keap1* deletion in macrophages resulted in reduced levels of inflammatory mediators, organ injury, bacteremia and mortality, whereas Nrf2 deletion had the opposite effects^27^. We found that KEAP1 plays an important role in macrophage pro-inflammatory activity through a pooled CRISPR screen of metabolic genes. Our study elucidated that *KEAP1* deletion inhibited whereas *ACOD1* deletion promoted macrophage pro-inflammatory activity through regulating NRF2. The expression of itaconate was abrogated by *ACOD1* deletion in *ACOD1*^-/-^ MSLN-CAR-iMACs. Itaconate is known to alkylate cysteine residues on KEAP1, promoting the accumulation and nuclear translocation of NRF2, which leads to the expression of downstream genes with anti-oxidant and anti-inflammatory properties. *ACOD1*^-/-^ MSLN-CAR-iMACs showed lower expression of NRF2, but higher levels of pro-inflammatory cytokines and ROS. *ACOD1*^-/-^ MSLN-CAR-iMACs exhibited enhanced M1-like polarization and stronger anti-tumor activity (Supplementary Fig. 13). We used screens in both tMACs and iMACs, and the limitation for the screen in iMACs was that many metabolic genes are necessary for macrophage differentiation and survival, and thus the essential genes will be missed in the list of positive candidates. In our case, only several genes were identified from the iMAC screen, including *ACOD1*. The reason that *ACOD1* can be picked up might be that its expression is not required in unstimulated macrophages or during macrophage differentiation, and it is only induced upon LPS + IFN-γ stimulation. Thus it is not considered as an essential gene. To obtain more candidates, an inducible system of CRISPR screen would be a better choice in which Cas9 can be induced after differentiation^28–30^. Besides *ACOD1*, many other highly ranked genes coming out of our metabolic screen may also contribute to macrophage pro-inflammatory activity, such as *ULK1*, *GCLM*, *PPARD*, *GPD2*, and so on. GPD2 regulates LPS-induced macrophage tolerance via a pathway distinct from ACOD1^31^. Therefore, the double knockout of two genes that work in orthogonal pathways may represent another metabolic engineering strategies to further enhance macrophage functions in cancer immune cell therapies.

ACOD1 plays a crucial role in mitochondrial metabolism, which is tightly connected to many aspects of cellular functions. We also observed the maximal oxygen consumption was decreased in *ACOD1*^-/-^ iMACs. ACOD1 produces itaconate in response to pathogen infection and inflammation^15^. Itaconate can inhibit inflammasome activation^32,33^ or regulate immune tolerance through succinate dehydrogenase (SDH) in macrophages^34–36^. For instance, Lampropoulou et.al showed itaconate could inhibit SDH and resulted in increased succinate level, and Irg1 deletion led to abrogation of succinate accumulation^36^, and Chen et.al showed overexpression of Irg1, but not its catalytically inactive mutant, results in elevated intracellular levels of itaconate and succinate^37^. Thus we can not exclude that *ACOD1* deletion might influence the macrophage polarization phenotype through other downstream metabolites.

Since ACOD1 and itaconate have important roles in the anti-inflammatory effects of macrophages, most previous studies focused on their functions in infectious diseases^37,38^, However, limited studies have shown the role of human ACOD1 in immune-oncology and its possible applications in myeloid cell-based adoptive cell transfer in cancer immunotherapy. Engineered iMACs such as CAR-iMACs provide a platform for cancer immune cell therapy^9,39^. In this study, we demonstrated ACOD1 deficiency could promote stronger anti-solid tumor function than wild type MSLN-CAR-iMACs. We have also shown that *ACOD1*^-/-^ MSLN-CAR-iMACs combined with low dose ICIs could further elevated anti-ovarian cancer capacity. Thus, ACOD1 is a fresh metabolic target to engineer CAR-iMACs, in order to elevate their anti-tumor function and to eliminate tumor cells.

Macrophages can both kill cancer cells and modulate the TME depending on their phagocytosis function and pro-inflammatory activity^40^, which can be greatly enhanced by *ACOD1* deletion as we have shown above in this work. Our results also showed that the iMAC-tumor co-culture supernatant alone indeed had tumor killing function as well. Either cytokines with cytolytic activity or ROS in the supernatant could confer the function. Neutralizing antibodies of TNF-α and IFN-γ did not abrogate tumor killing function of the supernatant (Fig. 6f). However, we could not completely rule out there could be other cytokines that mediated the cytolytic activity. Supplementing NAC reversed the phenotype, suggesting ROS contributed to the tumor killing function. Regarding indirect functions through other immune cells, as our experiments were conducted either in the absence of other immune cell types in vitro, or based on immuno-deficient NSG mice, which do not have the endogenous NK and T cells, the tumor killing activity in these settings was unlikely through stimulating NK and T cells. We can’t exclude that in a humanized model, the engineered CAR-iMAC cells may also influence the other endogenous immune cells to confer their anti-tumor activity. Overall, our current data support a multi-level mechanism of CAR-iMACs in tumor killing activity, including enhanced direct phagocytosis and more ROS produced by *ACOD1* knockout.

Cytokines secreted from highly proliferative immune cells might lead to cytokine release syndrome. However, as macrophages do not have the capacity to proliferate in vivo, the amount of secreted cytokines might not reach to a level that can lead to toxicity in patients. Nevertheless, the effect of increased pro-inflammatory cytokines in *ACOD1*^-/-^ macrophages in vivo merits further investigations using humanized models to provide guidance for choosing the optimal dose in clinical research in the future.

## Methods

### Ethical approval

All the mice were maintained under a specific pathogen-free (SPF) condition in the animal facility of Zhejiang University. Carbon dioxide was used for euthanasia. All the animal experiments were strictly conducted in accordance with the protocols approved by the the Animal Research Committee guidelines of Zhejiang University.

### Cell Lines

HEK293T (ATCC CRL-3216), THP-1 (ATCC TIB-202), K562 (ATCC CCL-243), Nalm6 (ATCC CRL-3273) and AsPC-1 (ATCC CRL-1682) were obtained from the National Collection of Authenticated Cell Cultures and cultured according to standard protocols. HO-8910 (MZ-0089) was purchased from NingboMingZhoubioCO.,Ltd (Zhejiang, China). Human iPSCs were obtained from the reprogramming of peripheral blood mononuclear cells from a volunteer donor, as described before^10^. The experiment was approved by the Human Subjects Committee of Jinling Hospital, Nanjing University. Approval number: 2020DZSKTZX-007. Human iPSCs were cultured in mTeSR medium (85852, STEMCELL Technologies) with Matrigel Matrix (354277, Corning) coated plates.

### Plasmid construction and single guide RNA cloning

All the Cas9-expressing THP-1 cells or iPSC lines in this study were derived by lentiviral transduction with a Cas9 expression vector containing an optimized sgRNA backbone (LentiCRISPR v2; Addgene, 52961). All of the sgRNAs were cloned into the LentiCRISPR v2 vector following the protocol described before^41^. And the sgRNAs used in this study were shown in Supplementary Table 1. The annealed sgRNA oligonucleotides were ligated with T4 DNA ligase (M0569S, NEB) to the BsmB1-digested LentiCRISPR v2 vector.

### Lentivirus production

We produced lentivirus using HEK293T cells cultured in DMEM supplemented with 1% penicillin-streptomycin and 10% FBS. The CRISPR library vectors (Human CRISPR Metabolic Gene Knockout Library; Addgene, Pooled Library #110066)^12^ or the single sgRNA vectors, envelop vector pMD2.G, and packaging vector psPAX2 were mixed in a 4:3:1 ratio in OPTI-MEM (Thermo Fisher Scientific, 31985070) and Polyethylenimine (PEI) (Polysciences, 9002-98-6), and transfected into HEK293T cells at 80% to 90% confluence in 10-cm tissue culture plates. The supernatant was collected at 24, 48, and 72 h post-transfection, filtered via a 0.45 μm filtration unit (Millipore, Cat# SLHVR33RB), and mixed overnight at 4 °C with one-third volume of 30% PEG8000. The medium was concentrated at 3200 × g for 30 min at 4 °C. The pellet was resuspended in PBS and stored at -80 °C.

### Transduction of lentivirus containing sgRNAs

For transfection of THP-1 cells and iPSCs, we infected cells with lentivirus and 5 μg/mL polybrene overnight, and the medium was changed the following day. After puromycin (1 μg/mL for THP-1 cells and 250 ng/mL for iPSC) selection for seven days, >95% of the population was transfected, and the cells were ready to be used for the subsequent experiments.

### Pooled CRISPR screen

1.5 × 10^7^ THP-1 cells were transduced with a viral library for 24 h (MOI = 0.3). After puromycin (1 μg/mL) selection for seven days, 1.5 × 10^7^ transduced cells were collected as input samples. The other transduced cells were treated with PMA (50 ng/mL) for 48 h, then stimulated by LPS (50 ng/mL) plus IFN-γ (50 ng/mL) for 24 h. The stimulated cells were harvested and stained with PE anti-human CD80 (Biolegend, Cat:305208, Clone: 2D10, Lot: B330518) for 15 min at room temperature. The CD80^high^ and CD80^low^ cells were separated by flow cytometry sorting. The genomic DNA of cells was isolated, and the sgRNA library was barcoded and amplified for two rounds of PCR. PCR products were purified for sequencing on an Illumina HiSeq 2500. The sequencing data was analyzed by MAGeCK^42^.

### Generation of CRISPR/Cas9 knockout cells

LentiCRISPR v2 vectors targeting *KEAP1* and *ACOD1* were constructed as described before^41^. The THP-1 cells and iPSC were infected with lentivirus expressing Cas9 and sgRNAs targeting *KEAP1* and *ACOD1*. After puromycin selection for seven days, the THP-1 cells were expanded, and knockout efficiency was verified using qPCR and western blotting. After puromycin selection for three days, iPSCs were passaged, and the clones grown from single cells were picked up and expanded. The knockout efficiency of iPSC was verified by sequencing, qPCR, and western blot analyses.

### Derivation of iMACs from iPSCs

The derivation of iMAC from iPSCs has been previously described^10^. Briefly, 8000 iPSCs were seeded in 96-well round-bottom plates with APEL2 medium (05271, STEMCELL Technologies) containing 100 ng/mL human Stem Cell Factor (SCF), 50 ng/mL human Vascular Endothelial Growth Factor (VEGF), 10 ng/mL recombinant human Bone Morphogenetic Protein 4 (BMP-4), 5 ng/mL human FGF-basic (154 a.a.), and 10 mM Rho kinase inhibitor (ROCK inhibitor, Y27632, Sigma). After eight days of hematopoietic differentiation, spin embryoid bodies (EB) were transferred into Matrigel-coated 6-well plates under macrophage differentiation conditions. Macrophage differentiation medium is StemSpan-XF (100-0073, STEMCELL Technologies) containing 10 ng/mL human FGF-basic (154 a.a.), 50 ng/mL human Vascular Endothelial Growth Factor (VEGF), 50 ng/mL human Stem Cell Factor (SCF), 10 ng/mL recombinant human Insulin-like Growth Factor-1 (IGF1), 20 ng/mL IL-3, 50 ng/mL recombinant human M-CSF, and 50 ng/mL recombinant human GM-CSF. The floating cells were collected from the supernatant and directly transferred into uncoated 6-well plates in macrophage culture medium. The macrophage culture medium is StemSpan-XF containing 50 ng/mL recombinant human M-CSF and 50 ng/mL recombinant human GM-CSF.

### Flow cytometry

The tMACs or iMACs were stimulated with LPS and IFN-γ for the indicated time. The single-cell suspensions were then prepared and incubated with an antibody or antibody cocktails for 15 min at room temperature for cell surface staining. Antibodies used in this study were PE Mouse IgG1, κ isotype (Biolegend, Cat: 400113, Clone: MOPC-21, Lot: B245984), APC Mouse IgG1, κ isotype (Biolegend, Cat: 400119, Clone: MOPC-21, Lot: B243042), FITC Mouse IgG1, κ isotype (Biolegend, Cat: 400107, Clone: MOPC-21, Lot: B199152), APC anti-human CD206 (Biolegend, Cat: 321109, Clone: 15-2, Lot: B348965), APC anti-human CD86 (Biolegend, Cat: 305411, Clone: IT2.2, Lot: B351349), PE anti-human CD80 (Biolegend, Cat:305208, Clone: 2D10, Lot: B330518), PE anti-human CD163 (Biolegend, Cat: 333606, Clone: GHI/61, Lot: B347256), FITC anti-human CD14 (Biolegend, Cat: 325604, Clone: HCD14, Lot: B268830) and APC anti-humanCD11B (Biolegend, Cat: 301309, Clone: ICRF44, Lot: B278346). These antibodies were used at a 1:100 dilution. Data were recorded on Beckman DxFLEX and analyzed with the FlowJo V10 software.

### In vitro tumor killing assay

The functionality of CAR-iMACs was assessed by co-culturing them with luciferase-expressing tumor cells in a 96-well plate (WHB-96-2). CAR-iMACs and 2 × 10^3^ tumor cells were mixed in a total volume of 100 μL RPMI 1640 complete medium, and the number of cells was determined based on the indicated E:T ratio. After co-culturing for 24 or 48 h, 25 μL of 33 mg/mL D-Luciferin (GoldBio, 115114-35-9) was added to each well, and luminescent signals were analyzed using a microplate reader (TECAN, SPARK).

For the 4-OI supplement assay, iMACs were pre-treated with 4-OI (MCE, HY-112675, 250 μM) or DMSO (Sigma-Aldrich, 41639) control for 3 h before challenging them with LPS (InvivoGen, tlrl-eblps) plus human IFN-γ (PeproTech, 300-02-100UG) (50 ng/mL each). The iMACs were then co-cultured with luciferase-expressing tumor cells.

To test the function of NRF2, SFN (MCE, HY-13755, 10 μM) was added to the co-culture system.

To test the function of cytokines, the supernatant was collected after iMACs were co-cultured with HO-8910 cells for 24 h (E:T = 10:1). Then human IgG1 isotype control (BioXcell, BE0297), neutralizing antibody (10 μg/mL) of IFN-γ (BioXcell, BE0235), or TNF-α (BioXcell, SIM0006) was added to the supernatant, and the cytotoxicity activity was measured by luciferase assay.

### Enzyme-linked immunosorbent assay

The supernatant of iMAC culture or tumor-iMAC co-culture was collected and centrifuged at 300 × g for 10 min to remove the precipitate. Human IL-6, IL-1β, CXCL-10, IFN-γ and TNF-α were quantified using Elisa kits (MultiSciences, EK106, EK101B, EK168, EK180, EK182) following the manufacturer’s protocols.

### In vivo anti-tumor assay

For in vivo experiments, 6–8-week-old NOD/SCID/IL2rγnull (NSG) mice (Stock No: 005557) (The Jackson Laboratory, Bar-Harbor, Maine, USA) were maintained under pathogen-free conditions under the Zhejiang University Institutional Animal Care and followed the committee’s approved protocols. All mice were maintained in suitable temperature (25 °C) and humidity environment (typically 50%), 12 h dark/light cycle, and fed with sufficient water and food. The catalogue number of the diet is 1010085 (Xietong).

Mice were sacrificed when the average tumor diameter exceeds 20 mm in mice or when tumor volumes exceeded 2000 mm^3^. Mice will also be sacrificed when the tumor metastasizes or rapidly grows to the point of ulceration, causing infection or necrosis. For survival studies, mouse were monitored carefully and tumor burdens were measured once a week after initial inoculation.

In the first ovarian cancer mouse model, 6–8-week-old female NSG mice were used, 2 × 10^5^ luciferase gene expressing HO-8910 cells were inoculated IP before treatment (day -4). After tumor cell inoculation, mice were randomly assigned to experimental groups (n = 5 per group). Four days later, 4 × 10^6^ MSLN-CAR-iMACs or *ACOD1*^-/-^ MSLN-CAR-iMACs were inoculated IP (day 0) for therapy. The tumor burden was determined by BLI using an In Vivo Imaging system (IVIS) Imaging System (BiospaceLab photonimager).

In the ovarian cancer orthotopic injection mouse model with CAR-iMAC and anti-CD47 antibody combined therapy, 6–8-week-old female NSG mice were used, 1 × 10^5^ luciferase gene expressing HO-8910 cells were inoculated directly into ovary before treatment (day -4). After tumor cell inoculation, mice were randomly assigned to experimental groups (n = 5 per group). Four days later, mice received a single in situ injection of 6 × 10^6^ MSLN-CAR-iMAC or *ACOD1*^-/-^ MSLN-CAR-iMACs (day 0) combined with a low-dose CD47 antibody (50 μg/mouse, twice a week) for therapy. Tumor burden was determined by BLI.

In ovarian cancer mouse model with CAR-iMAC and the anti-PD1 antibody (Sintilimab) (Chemstan, Cat: CSD00572) combined therapy, 6–8-week-old female NSG mice were used, 2 × 10^5^ luciferase gene expressing HO-8910 cells were inoculated IP before treatment (day -4). After tumor cell inoculation, mice were randomly assigned to experimental groups (n = 5 per group). Four days later, mice received a single injection of 4 × 10^6^ MSLN-CAR-iMAC or *ACOD1*^-/-^ MSLN-CAR-iMACs IP (day 0) combined with a low-dose anti-PD1 antibody (100 μg/mouse, twice a week) for therapy. The tumor burden was determined by BLI using an IVIS Imaging System (BiospaceLab photonimager).

In the pancreatic cancer mouse model, 6–8-week-old male NSG mice were used. 1 × 10^5^ AsPC−1 cells were inoculated IP before treatment (day -4). After tumor cell inoculation, mice were randomly assigned to experimental groups (n = 5 per group). AsPC-1 cells grow fast in vivo, in order to get a better therapeutic effect, a higher E:T ratio was used. Four days later, 1.5 × 10^7^ MSLN-CAR-iMACs or *ACOD1*^-/-^ MSLN-CAR-iMACs were IP injected (day 0). The tumor burden was determined by BLI later.

### Western blotting

Pellets from 1 × 10^6^ cells were collected and resuspended with 100 μL RIPA Buffer (Beyotime, Cat: #P0013J). The samples were incubated on ice for 30 min and centrifuged at 15000×g for 15 min at 4 °C. The supernatant was collected, and the protein concentration was measured by BCA analysis (Thermo Scientific, Cat: #23225). Approximately 50 μg of total protein was loaded for western blotting. Antibodies used in this study were HRP AffiniPure Goat anti-Rabbit IgG (H + L) secondary antibody (EARTHOX, Cat: 620822, 1:2000), β-Actin (13E5) Rabbit mAb (Cell Signaling Technology, Cat: #4970, 1:1000), NRF2 (D1Z9C) Rabbit mAb (Cell Signaling Technology, Cat: #12721, 1:1000), Anti-Keap1 antibody (abcam, Cat: ab227828, 1:1000), Anti-IRG1 antibody (abcam, Cat: ab222411, 1:1000). Representative images were obtained using the ChemiDoc Touch Imaging System (Bio-Rad).

### Real-time reverse transcription-PCR

RNA was extracted from macrophages or tumor cells using Total RNA Isolation Kit V2 (Vazyme, Cat# RC112-01). Reverse transcription from RNA to cDNA use Hiscript Reverse Transcriptase (Vazyme, Cat# R302-01). PCR reactions were performed on a CFX96 Real-Time PCR System (Bio-Rad Laboratories) using ChamQ Universal SYBR qPCR Master Mix (Vazyme, Cat# Q711-02). All the primers for qRT-PCR used in this study were shown in Supplementary Table 2.

### Metabolic studies

OCR was measured by Seahorse XFe96 Analyzer (Agilent) using a Seahorse XF Cell Mito Stress Test Kit (Agilent, 103015-100). iMACs were resuspended in an RPMI1640 medium containing LPS (50 ng/mL) plus IFN-γ (50 ng/mL) and then seeded at 5 × 10^4^ cells/well in an XF96 plate. Eight hours later, the RPMI 1640 medium was changed to XF RPMI medium. The OCR was measured (pmol/min) real-time in an XFe96 Extracellular Flux Analyzer. iMACs were stimulated with LPS and IFN-γ for 24 h, and the OCR was in response to 1.5 μM oligomycin, 2 μM fluorcarbonylcyanide phenylhydrazone (FCCP) and 500 nM rotenone and antimycin A. Basal OCR, MRC, ATP linked respiration, and mitochondrial spare respiratory capacity (SRC) was calculated by WAVE V2.6 software.

### RNA-seq

Total RNA was isolated and purified using FastPure Cell/Tissue Total RNA Isolation Kit V2 (Vazyme, RC112-01) from 2 × 10^6^ tMACs according to the manufacturer’s protocol. RNA qualification was performed using Nanodrop to check RNA purity (OD260/0D280) and Agilent 2100 to check RNA integrity. A total amount of 2 μg RNA per sample was used for RNA-seq libraries preparation. RNA-seq libraries were prepared using VAHTS Stranded mRNA-seq Library Prep Kit for Illumina V2 (Vazyme, NR612-02) according to the manufacturer’s protocol and sequenced on an Illumina Hiseq 2500. The threshold of differentially expressed genes is p-adj <0.05. The color descending from red to blue in the heatmaps of differentially expressed genes indicated log10 (FPKM + 1) from large to small.

### Gene set enrichment analysis

To identify biological signatures depleted or enriched following CD80-based sorting or in the *KEAP1* knockout macrophages, we used DAVID Bioinformatics Resources (https://david.ncifcrf.gov/). We focused on the biological oncology of the GO gene sets to obtain the indicated enrichment score.

### Statistical analysis

All data are presented as mean ± SD. Comparisons between different groups were analyzed by the one-way analysis of variance (ANOVA), two-way analysis of variance (ANOVA), and unpaired two-tailed Student’s *t*-test. Kaplan-Meier survival curves were compared with the log-rank test. Statistical analyses were performed in GraphPad Prism 9.0.0 software using the statistical tests indicated for each experiment. All tests were considered significant at p < 0.05.

### Reporting summary

Further information on research design is available in the Nature Portfolio Reporting Summary linked to this article.

### Supplementary information

Supplementary Information

Peer Review File

Reporting Summary

### Source data

Source Data

## Data Availability

The data used to generate the main results are shown in main Figs and Supplementary Figs are available as supplementary information. Source data include uncropped western blots. All data supporting the findings of this study are available in a publicly accessible repository. The RNA-seq data that support the findings of this study have been deposited in the Gene Expression Omnibus (GEO) under the following accession codes: GSE216352. The pooled screen data have been deposited in GEO under the accession number: GSE216353. Both are under the accession number: GSE216354. Source data are provided with this paper.
