# Supplementary material for: Metabolic Reprogramming via ACOD1 depletion enhances function of human induced pluripotent stem cell-derived CAR-macrophages in solid tumors
Source: Nat Commun. 2023 Sep 18;14:5778. doi: 10.1038/s41467-023-41470-9 (PMC10507032; doi:10.1038/s41467-023-41470-9)
Supplement: Supplementary file 1 — Supplementary Information [file 41467_2023_41470_MOESM1_ESM.pdf]

Supplementary Information

Metabolic Reprogramming via ACOD1 depletion enhances function of human induced pluripotent stem cell-derived CAR-macrophages in solid tumors

Xudong Wang[1,2,#], Siyu Su[2,3,#], Yuqing Zhu[1,4], Xiaolong Cheng[5,6], Chen Cheng[1], Leilei Chen[7], Anhua Lei[1,8], Li Zhang[1], Yuyan Xu[1], Dan Ye [7,9], Yi Zhang[3], Wei Li[5,6], Jin Zhang[1,2,10,11,*].

[1]Center for Stem Cell and Regenerative Medicine, Department of Basic Medical Sciences, and Bone Marrow Transplantation Center of the First Affiliated Hospital, Zhejiang University School of Medicine, Hangzhou, 310003, China;
[2]Liangzhu Laboratory, Zhejiang University, Hangzhou, 311121, China;
[3]Quanzhou First Hospital Affiliated to Fujian Medical University, Quanzhou 362000, China;
[4]Center for Stem Cell and Translational Medicine, School of Life Sciences, Anhui University, Hefei, Anhui 230601, P.R. China
[5]Center for Genetic Medicine Research, Children's National Hospital, 111 Michigan Ave NW, Washington, DC, 20010, USA;
[6]Department of Genomics and Precision Medicine, George Washington University, 111 Michigan Ave NW, Washington, DC, 20010, USA;
[7]Shanghai Key Laboratory of Clinical Geriatric Medicine, Shanghai, Huadong Hospital, and Shanghai Key laboratory of Medical Epigenetics, International Co-laboratory of Medical Epigenetics and Metabolism (Ministry of Science and Technology), and Molecular and Cell Biology Lab, Institutes of Biomedical Sciences, Shanghai Medical College of Fudan University, Shanghai, 200032, China;
[8]CellOrigin Inc., Hangzhou, 310000, China;
[9]Department of General Surgery, Huashan Hospital, Fudan University, Shanghai 200040, China;
[10]Institute of Hematology, Hangzhou, 310058, China;
[11]Center of Gene/Cell Engineering and Genome Medicine of Zhejiang Province, Hangzhou, 310000, China.
[#]Equally Contributing Authors: Xudong Wang, Siyu Su
[*]Corresponding author: Jin Zhang , zhgene@zju.edu.cn

Content: Supplementary Figures 1-13
Supplementary Tables 1-2

Supplementary Figure 1

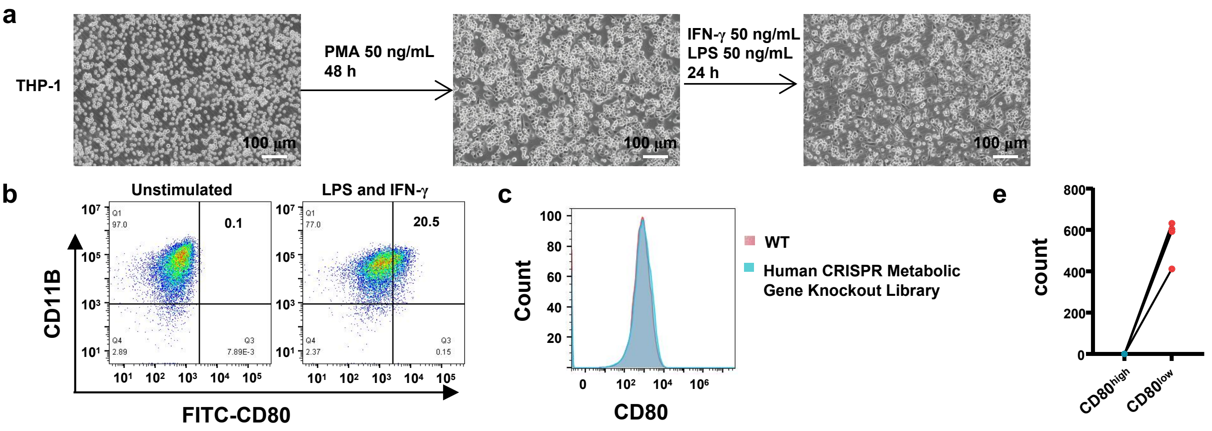

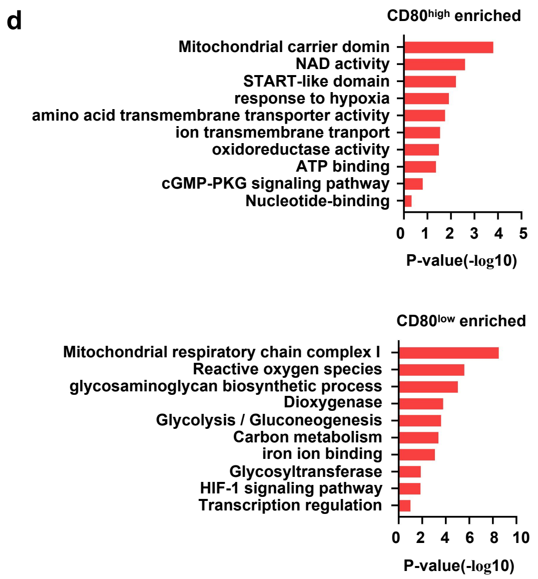

f

Gene	CD80^{low} | Rank
NDUFS6	1
MPST	2
CYB561A3	3
SLC4A7	4
KEAP1	5
NDST1	6
PPA2	7
LEPREL2	8
NDUFB5	9
OGFOD2	10
ATP5H	11
SLC16A13	12
NFKB1	13
PLPP4	14
ASMTL	15
PIGF	16
ATP5B	17
NR1H3	18
ACYP2	19
ALDH9A1	20

Gene	CD80^{high}| Rank
B3GNT8	1
ASNSD1	2
ST3GAL6	3
NANS	4
EPN2	5
CYP2R1	6
ULK1	7
PIP4K2A	8
GCLM	9
CECR1	10
TET3	11
MFSD3	12
ALG13	13
STARD13	14
ALDH2	15
PPARD	16
GPD2	17
AKR7A2	18
IMPDH2	19
GNPNAT1	20

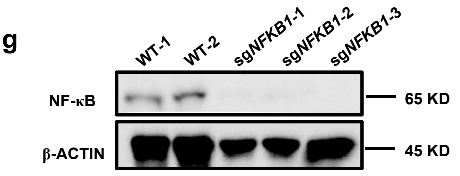

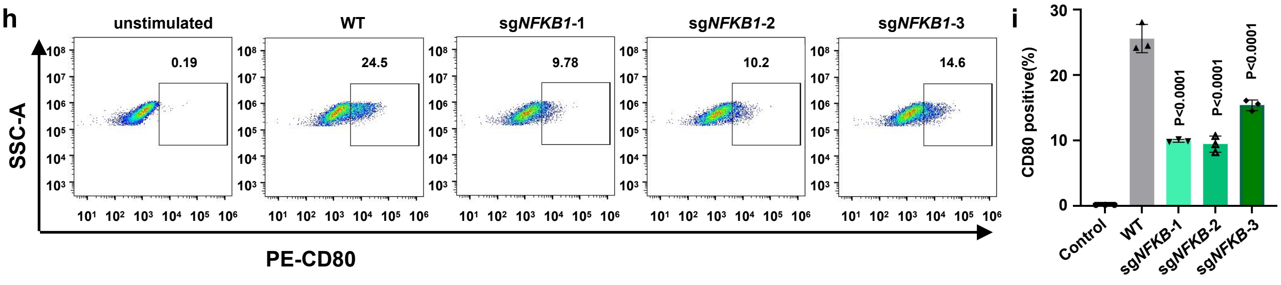

Supplementary Fig. 1 | Identifying the metabolic genes involved in human macrophage activation, related to **Fig. 1**. **a**, Microscopic pictures showing THP-1 cell differentiation and polarization. **b**, Flow cytometry plots and percentage of CD80 expression on WT THP-1-derived macrophages (tMAC) with or without LPS and IFN-γ stimulation for 24 h. **c**, WT and human Metabolic Gene CRISPR Library virus-infected THP-1 cells were differentiated into macrophages, and CD80 expression was measured by flow cytometry and demonstrated as histograms. **d**, GO term enrichment analysis with enriched sgRNA-targeted genes in the CD80^{high} population (up) and CD80^{low} population (down). **e**, Counts of sgRNAs targeting *KEAP1* detected in the CD80^{high} and CD80^{low} samples. **f**, Top 20 sgRNA-targeted genes enriched in the CD80^{low} populations and CD80^{high} populations identified by the CRISPR Screen in tMACs. **g**, The protein level of NF-κB in WT and *NFKB1*-depleted THP-1 cells. This experiment has been repeated for two times with similar results. **h,i**, Flow cytometry plots and quantification of CD80 expression on unstained, unstimulated, WT and *NFKB1*-depleted tMACs (**i**, n=3 biologically independent samples). Data was shown as mean ± SD. Statistics by one-way ANOVA test (WT vs sg*NFKB*-1, P<0.0001; WT vs sg*NFKB*-2, P<0.0001; WT vs sg*NFKB*-3, P<0.0001). Source data are provided as a Source Data file.

Supplementary Figure 2

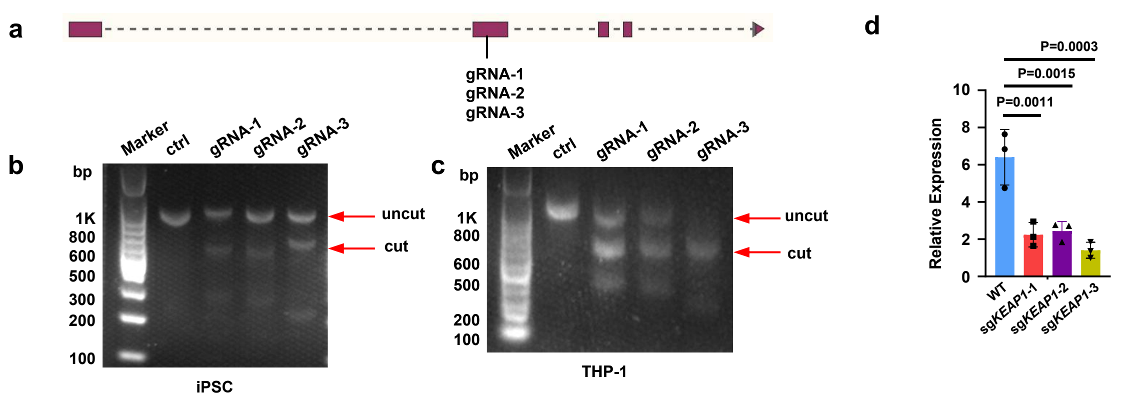

Supplementary Fig. 2 | *KEAP1* deletion in THP-1 cells, related to **Fig. 1**. **a**, CRISPR-Cas9-mediated *KEAP1* KO using three sgRNAs targeting exon 2 of the *KEAP1* gene. **b,c**, Validation of DNA cleavage efficiency by T7 endonuclease assays in iPSCs (**b**) and THP-1 cells (**c**). This experiment has been repeated for three times with similar results. **d**, Relative expression of *KEAP1* in WT and sg*KEAP1* transfected THP-1-derived macrophages (tMAC) (n=3 biologically independent samples). Data was shown as mean ± SD. Statistics by one-way ANOVA test (WT vs sg*KEAP1*-1, P=0.0011; WT vs sg *KEAP1*-2, P=0.0015; WT vs sg*KEAP1*-3, P=0.0003). Source data are provided as a Source Data file.

Supplementary Figure 3

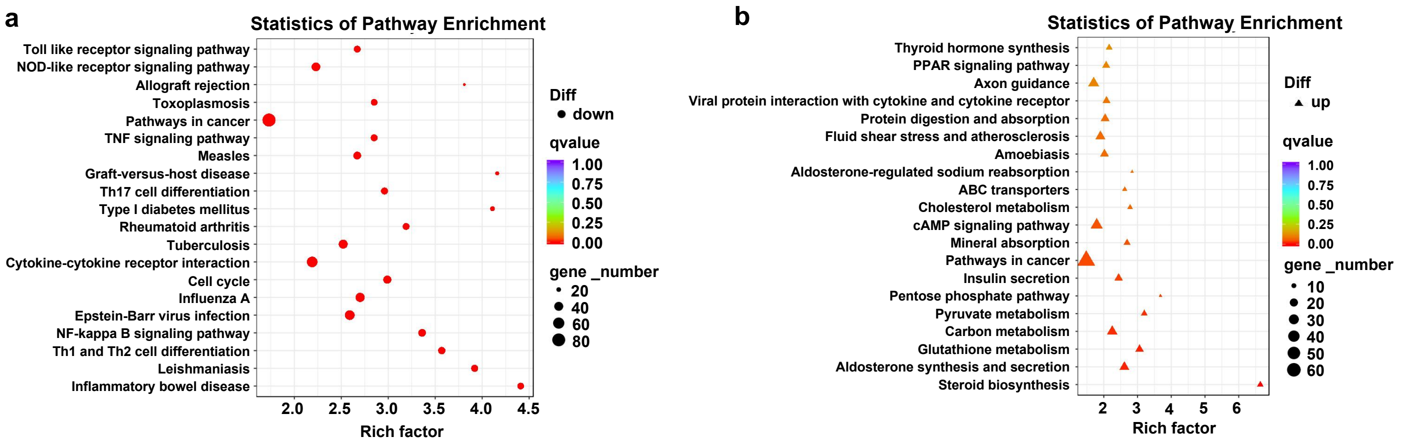

Supplementary Fig. 3 | Pathway enrichment in *KEAP1*-deleted macrophages, related to **Fig. 1. a,b**, Top enriched gene sets down-regulated (**a**) or up-regulated (**b**) in sg*KEAP1*-3-transduced THP-1-derived macrophages (tMAC) compared to sgControl-transduced cells after LPS and IFN-γ stimulation for 8 h (n=3 biologically independent samples).

Supplementary Figure 4

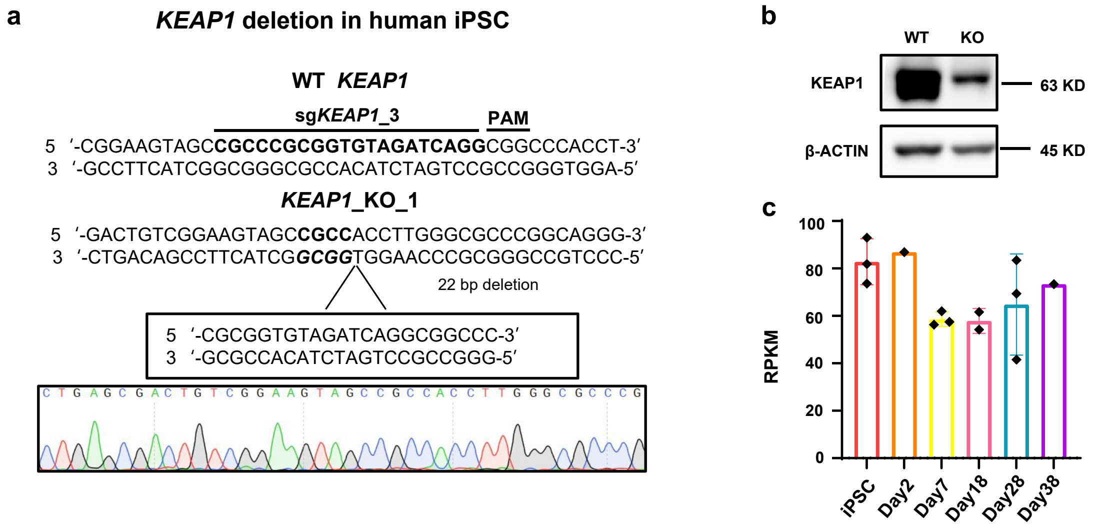

Supplementary Fig. 4 | *KEAP1* deletion in human iPSCs, related to **Fig. 1**. **a**, Comparison of the DNA sequence in the *KEAP1* KO iPSC clone (by Sanger sequencing) with *KEAP1* WT sequence, revealing a 22 bp deletion in the gRNA targeted region. **b**, The protein expression of *KEAP1* in WT and *KEAP1* deletion iPSCs was evaluated by western blotting. This experiment has been repeated for three times with similar results. **c**, RNA-seq data for the expression of *KEAP1* in iPSCs and differentiated cells on day 2, 7, 18, 28, and 38 (iPSC, n=3; Day 2, n=1; Day 7, n=3; Day 18, n=2; Day 28, n=3; Day 38, n=1; biologically independent samples). Data was shown as mean ± SD. Source data are provided as a Source Data file.

a

CD80high positive gRNA	rank
PIK3CA	1
PIGT	2
SFXN5	3
ALDH9A1	4
SLC17A9	5
SLC38A2	6
SCLY	7
GPX3	8
FOXRED2	9
GCLC	10
HADHA	11
ALDH18A1	12
NAA20	13
KDM6B	14
CBR4	15
DLST	16
SLC39A6	17
OXCT1	18
ACOD1	19
MFSD9	20

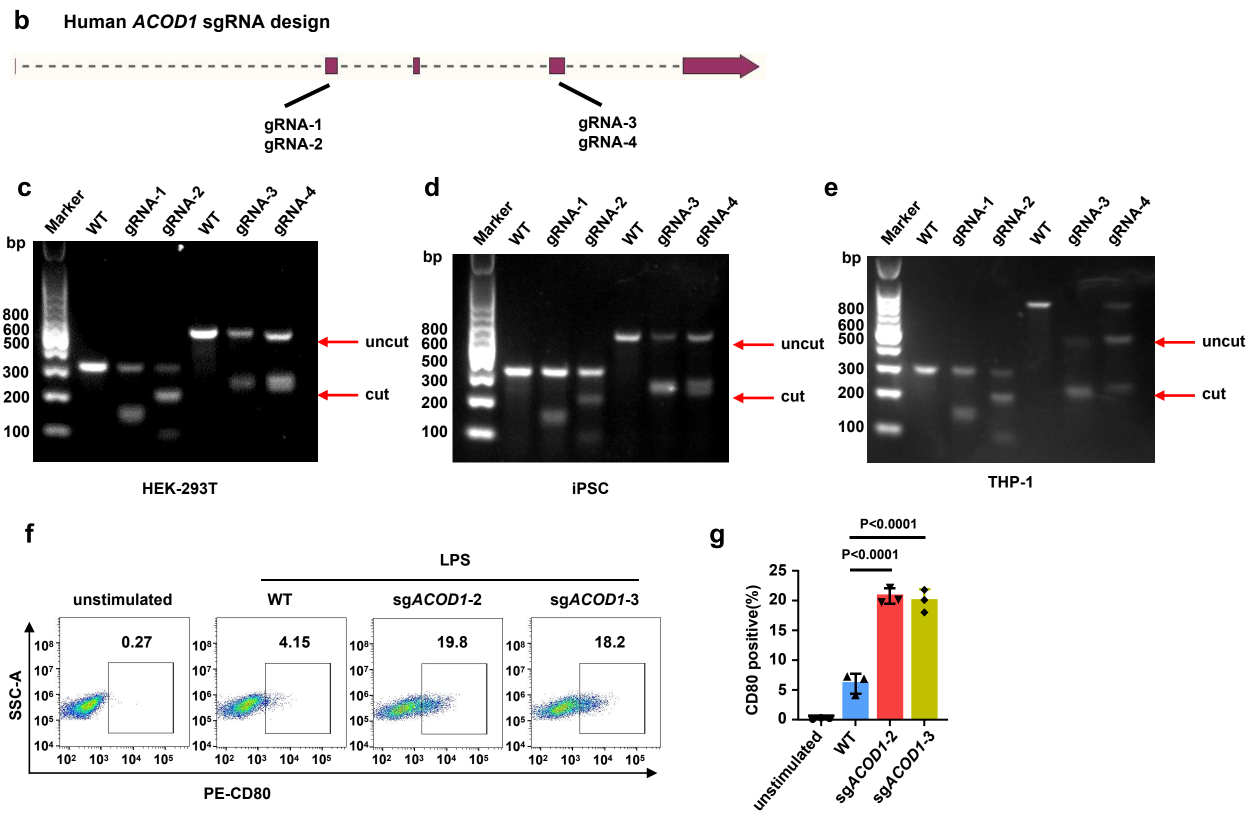

Supplementary Fig. 5 | *ACOD1* deletion in human macrophages resulted in enhanced pro-inflammation activation, related to **Fig. 2**. **a**, Top 20 sgRNA targeted genes enriched in the CD80^{high} population identified by a CRISPR Screen in iPSC-derived macrophages. **b-e**, CRISPR/Cas9-mediated *ACOD1* knockout using four sgRNAs located in exons 2 and 4 of the *ACOD1* gene, and validation of DNA cleavage efficiency by T7 endonuclease assays in HEK-293T (**c**), iPSC (**d**), and THP-1 cells (**e**). This experiment has been repeated for three times with similar results. **f,g**, Flow cytometry plots and quantification of CD80 expression in unstimulated, WT, and sg*ACOD1* transduced THP-1-derived macrophages (tMAC) with 50 ng/mL LPS stimulation for 24 h (**g**, n=3 biologically independent samples). Data was shown as mean ± SD. Statistics by one-way ANOVA test. (WT vs sg*ACOD1*-2, P<0.0001; WT vs sg*ACOD1*-3, P<0.0001) Source data are provided as a Source Data file.

Supplementary Figure 6

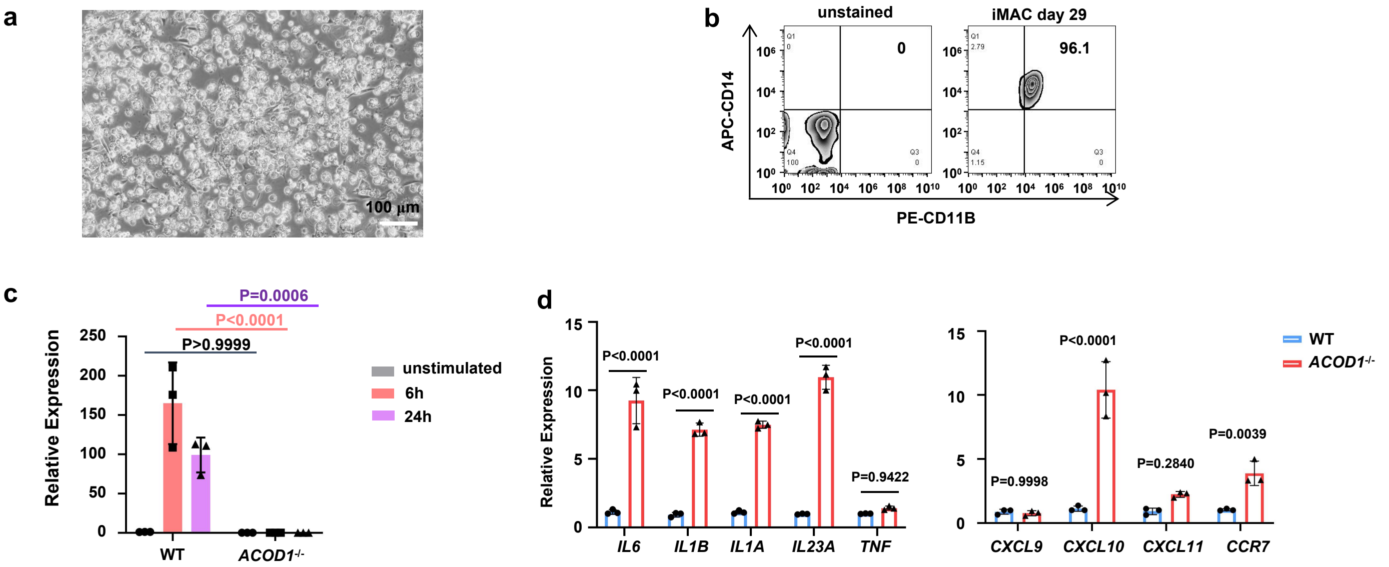

Supplementary Fig. 6 | *ACOD1* deletion resulted in elevated pro-inflammatory gene expression in iMACs, related to **Fig. 3**. **a**, Representative images of differentiated iMACs at day 29. This experiment has been repeated for greater than ten times with similar results. **b**, CD14 and CD11B expression on iMACs at day 29 was determined by flow cytometry. **c**, The relative expression of *ACOD1* in WT and *ACOD1*$^{-/-}$ iMACs with the indicated treatments, including unstimulated, and 50 ng/mL LPS plus 50 ng/mL IFN-γ stimulation for 6 and 24 h (n=3 biologically independent samples). Statistics by two-way ANOVA test. (unstimulated-WT vs unstimulated-*ACOD1*$^{-/-}$, P>0.9999; 6 h-WT vs 6 h-*ACOD1*$^{-/-}$, P<0.0001; 24 h-WT vs 24 h-*ACOD1*$^{-/-}$, P=0.0006) **d**, qRT-PCR for mRNA expression of pro-inflammatory genes and anti-inflammatory genes in WT and *ACOD1*$^{-/-}$ iMACs after LPS and IFN-γ stimulation for 24 h. (n=3 biologically independent samples) Statistics by two-way ANOVA test. *(IL6, IL1B, IL1A, IL23A*, P<0.0001; *TNF*, P=0.9422; *CXCL9*, P=0.9998; *CXCL10*, P<0.0001; *CXCL11*, P=0.2840; *CCR7*, P=0.0039) **c,d**, Data was shown as mean ± SD. Source data are provided as a Source Data file.

Supplementary Figure 7

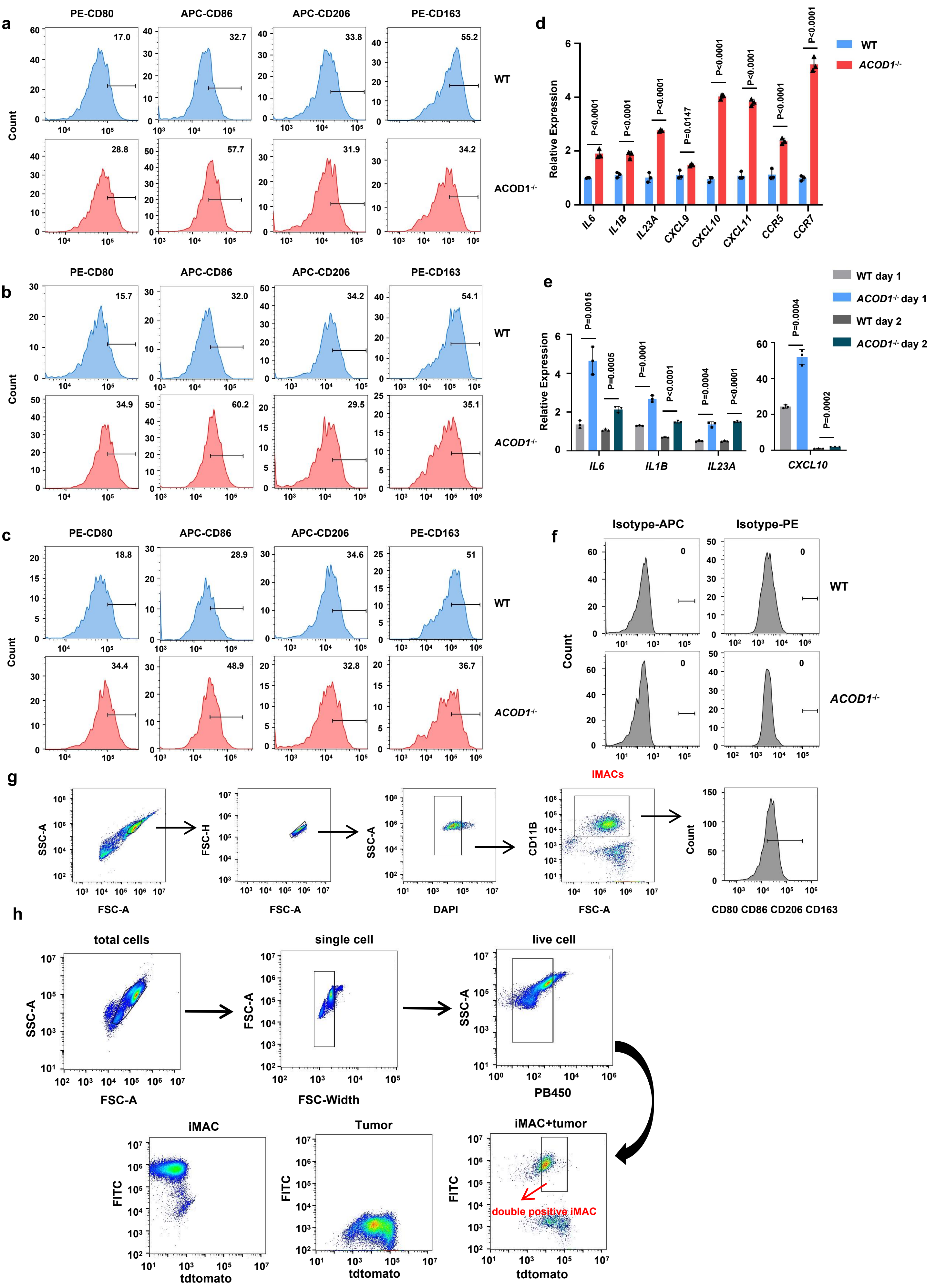

Supplementary Fig. 7 | *ACOD1*$^{-/-}$ iMACs exhibited increased pro-inflammatory activation when co-cultured with tumor cells, related to **Fig. 4**. **a-c**, The expressions of CD80, CD86, CD163, and CD206 in WT or *ACOD1*$^{-/-}$ iMACs after co-cultured with (**a**) Nalm6 (E:T=3:1), (**b**) K562 (E:T=5:1) or (**c**) K562 (E:T=3:1) for 24 h measured by flow cytometry and displayed as histograms. **d**, qRT-PCR for mRNA expression of pro-inflammatory genes in WT and *ACOD1*$^{-/-}$ iMACs after co-culture with Nalm6 (E:T=3:1) for 24 h (n=3 biologically independent samples). Data was shown as mean ± SD. Statistics by two-way ANOVA test. *(IL6, IL1B, IL23A*, *CXCL9*, *CXCL10*, *CXCL11*, *CCR5, CCR7*, P<0.0001) **e**, qRT-PCR for mRNA expression of pro-inflammatory genes in WT and *ACOD1*$^{-/-}$ iMACs after co-culture with Nalm6 (E:T=5:1) for 24 h (day 1) or 48 h (day 2) (n=3 biologically independent samples). Data was shown as mean ± SD. Statistics by two-way ANOVA test. *(IL6*: day 1, P=0.0015; day 2, P=0.0005. *IL1B*: day 1, P=0.0001; day 2, P<0.0001. *IL23A*: day 1, P=0.0004; day 2, P<0.0001. *CXCL10*: day 1, P=0.0004; day 2, P=0.0002.) **f**, WT or *ACOD1*$^{-/-}$ iMACs were stained by APC or PE isotype and displayed as histograms. **g**, Gating strategy of CD80-high, CD86-high, CD163-high, or CD206-high cells. **h**, Gating strategy of the phagocytosis assay. The iMACs were stained with a green dye and thus they were positive in the green channel, and the tumor cells were transduced with tdtomato, and thus there were positive in the red channel. The iMAC cells undergoing phagocytosis were those showing double positive, compared with the single positive iMACs or tumor cells. Source data are provided as a Source Data file.

Supplementary Figure 8

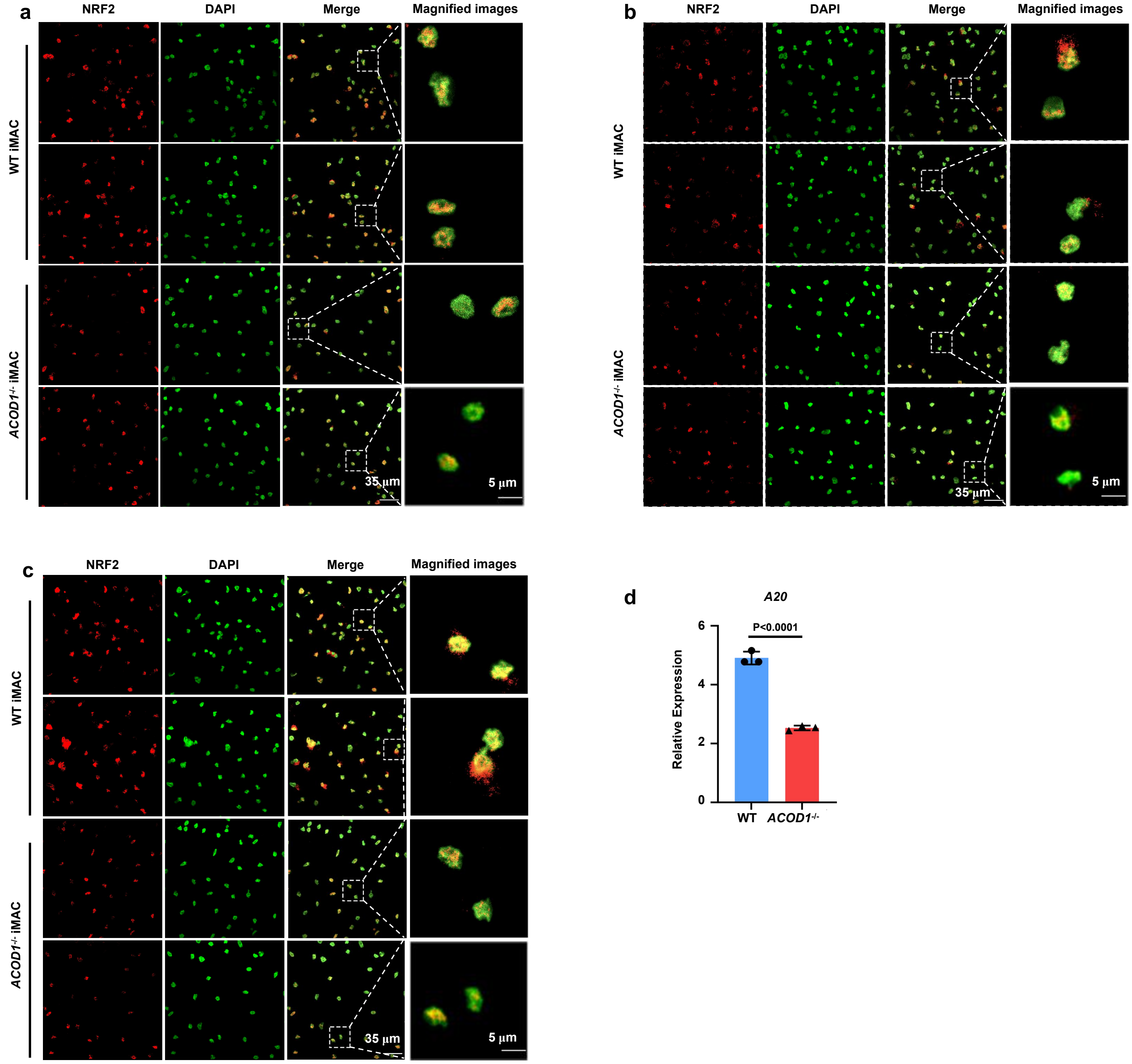

Supplementary Fig. 8 | *ACOD1* deletion resulted in decreased nuclear expression of NRF2 and decreased expression of the NF-κB pathway negative regulator *TNFAIP3* (*A20*), related to **Fig. 5**. **a-c**, Representative confocal images of NRF2 in WT and *ACOD1*$^{-/-}$ iMACs after LPS and IFN-γ stimulation for (**a**) 0 h, (**b**) 30 min, or (**c**) 8 h were obtained using the Olympus FV3000 microscope (n=60 cells from 3 biologically independent samples each group). This experiment has been repeated for three times with similar results. **d**, qRT-PCR for mRNA expression of *TNFAIP3* (*A20*) in WT and *ACOD1*$^{-/-}$ iMACs after LPS and IFN-γ stimulation for 24 h (n=3 biologically independent samples). Data was shown as mean ± SD. Statistics by unpaired t test (P<0.0001). Source data are provided as a Source Data file.

Supplementary Figure 9

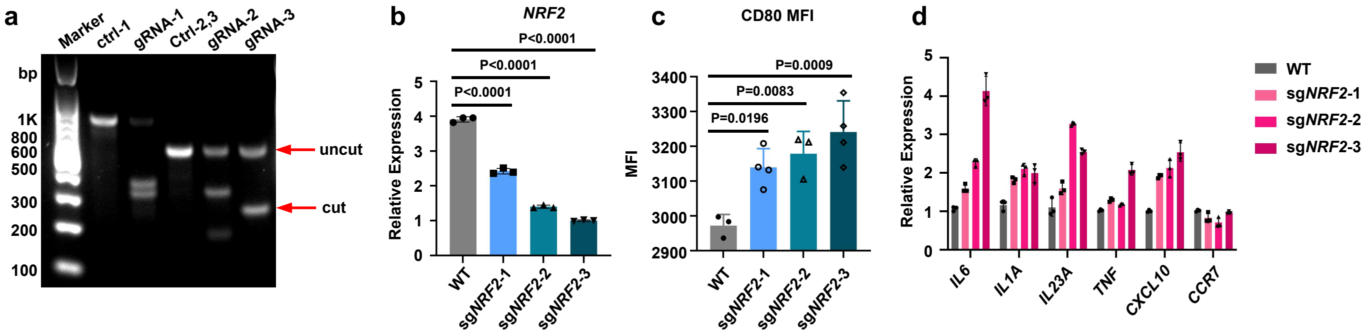

Supplementary Fig. 9 | *NRF2* deletion promoted pro-inflammatory activation in THP-1-derived macrophages, related to **Fig. 5**. **a**, Validation of DNA cleavage efficiency by T7 endonuclease assays in THP-1 cells. This experiment has been repeated for two times with similar results. **b**, qRT-PCR for mRNA expression of *NRF2* in WT and sg*NRF2*s transduced THP-1-derived macrophages (tMAC) (n=3 biologically independent samples). Statistics by one-way ANOVA test. (WT vs sg*NRF2*-1, P<0.0001; WT vs sg*NRF2*-2, P<0.0001; WT vs sg*NRF2*-3, P<0.0001) **c**, Quantification of CD80 mean fluorescence intensity (MFI) measured by flow cytometry in WT and sg*NRF2*s transduced tMACs after LPS and IFN-γ stimulation for 24 h (WT, n=3; sg*NRF2*-1, n=4; sg*NRF2*-2, n=3; sg*NRF2*-3, n=4 biologically independent samples). Statistics by one-way ANOVA test. (WT vs sg*NRF2*-1, P=0.0196; WT vs sg*NRF2*-2, P=0.0083; WT vs sg*NRF2*-3, P=0.0009) **d**, qRT-PCR for mRNA expression of pro-inflammatory genes in WT and sg*NRF2*s transduced tMACs after LPS and IFN-γ stimulation for 24 h (n=3 biologically independent samples). **b-d**, Data was shown as mean ± SD. Source data are provided as a Source Data file.

Supplementary Figure 10

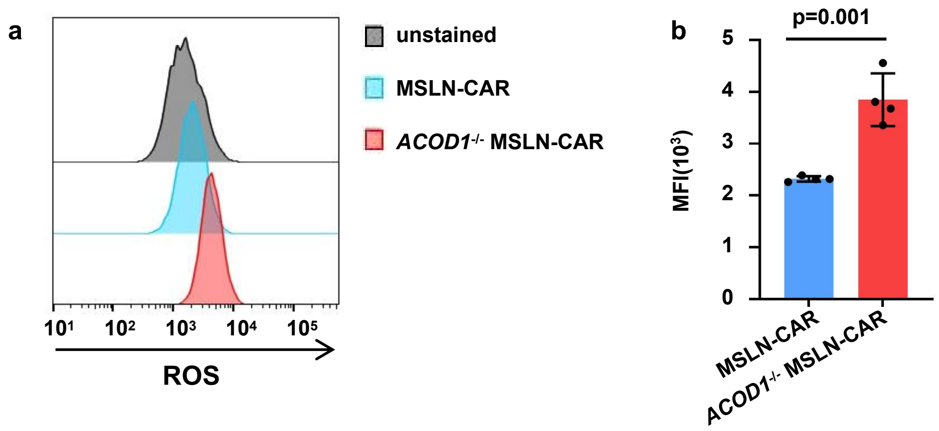

Supplementary Fig. 10 | *ACOD1* deletion promoted reactive oxygen species production in iMACs, related to **Fig. 6**. **a**,**b**, reactive oxygen species (ROS) in MSLN-CAR-iMACs and *ACOD1*$^{-/-}$ MSLN-CAR-iMACs (**a**) and mean fluorescence intensity (MFI) quantification (**b**) was determined by flow cytometry after stimulated by LPS plus IFN-γ (50 ng/mL each) for 24 h which were stained by MitoSOX Red Mitochondrial Superoxide Indicator. **b**, Data were shown as mean ± SD (n=4 biologically independent samples), Statistics by unpaired t test (P=0.001). Source data are provided as a Source Data file.

Supplementary Figure 11

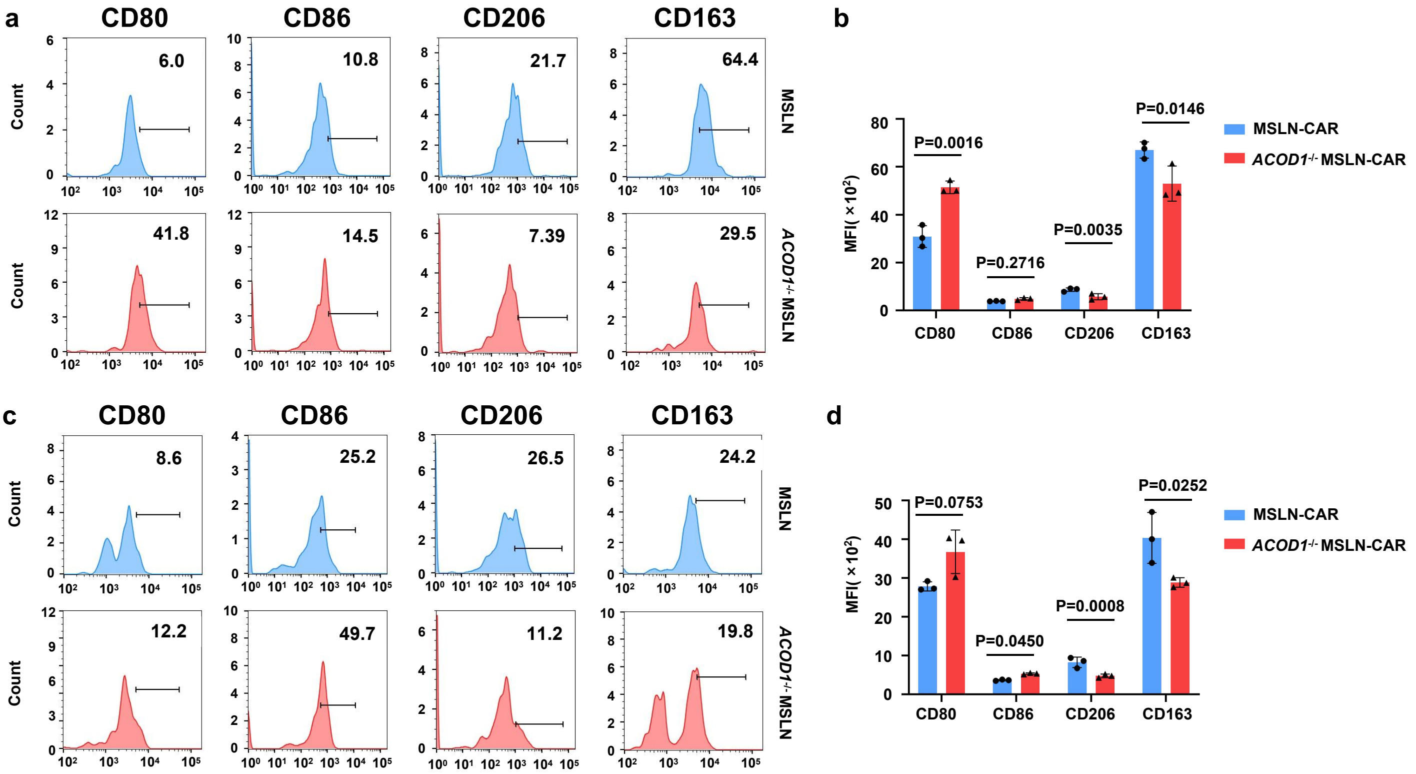

Supplementary Fig. 11 | *ACOD1* deletion promoted pro-inflammatory activity of iMACs in vivo, related to **Fig. 6**. **a**,**b,** Subcutaneous tumor model was established in NOD-scid IL2Rgnull (NSG) mice. 7 days later, MSLN-CAR-iMACs or *ACOD1*$^{-/-}$ MSLN-CAR-iMACs were injected intratumorally. After 7 days, the expression of CD80, CD86, CD206, and CD163 in MSLN-CAR-iMACs or *ACOD1*$^{-/-}$ MSLN-CAR-iMACs was measured by flow cytometry and the representative data was displayed as histograms. **b.** Data was shown as mean ± SD, n=3 biologically independent samples. Statistics by two-way ANOVA test. (CD80, P=0.0016; CD86, P=0.2716; CD206, P=0.0035; CD163, P=0.0146) **c**,**d,** After 14 days, the expression of CD80, CD86, CD206, and CD163 in MSLN-CAR-iMACs or *ACOD1*$^{-/-}$ MSLN-CAR-iMACs was measured by flow cytometry and the representative data was displayed as histograms. **d**, Data was shown as mean ± SD, n=3 biologically independent samples. Statistics by two-way ANOVA test. (CD80, P=0.0753; CD86, P=0.0450; CD206, P=0.0008; CD163, P=0.0252) Source data are provided as a Source Data file.

Supplementary Figure 12

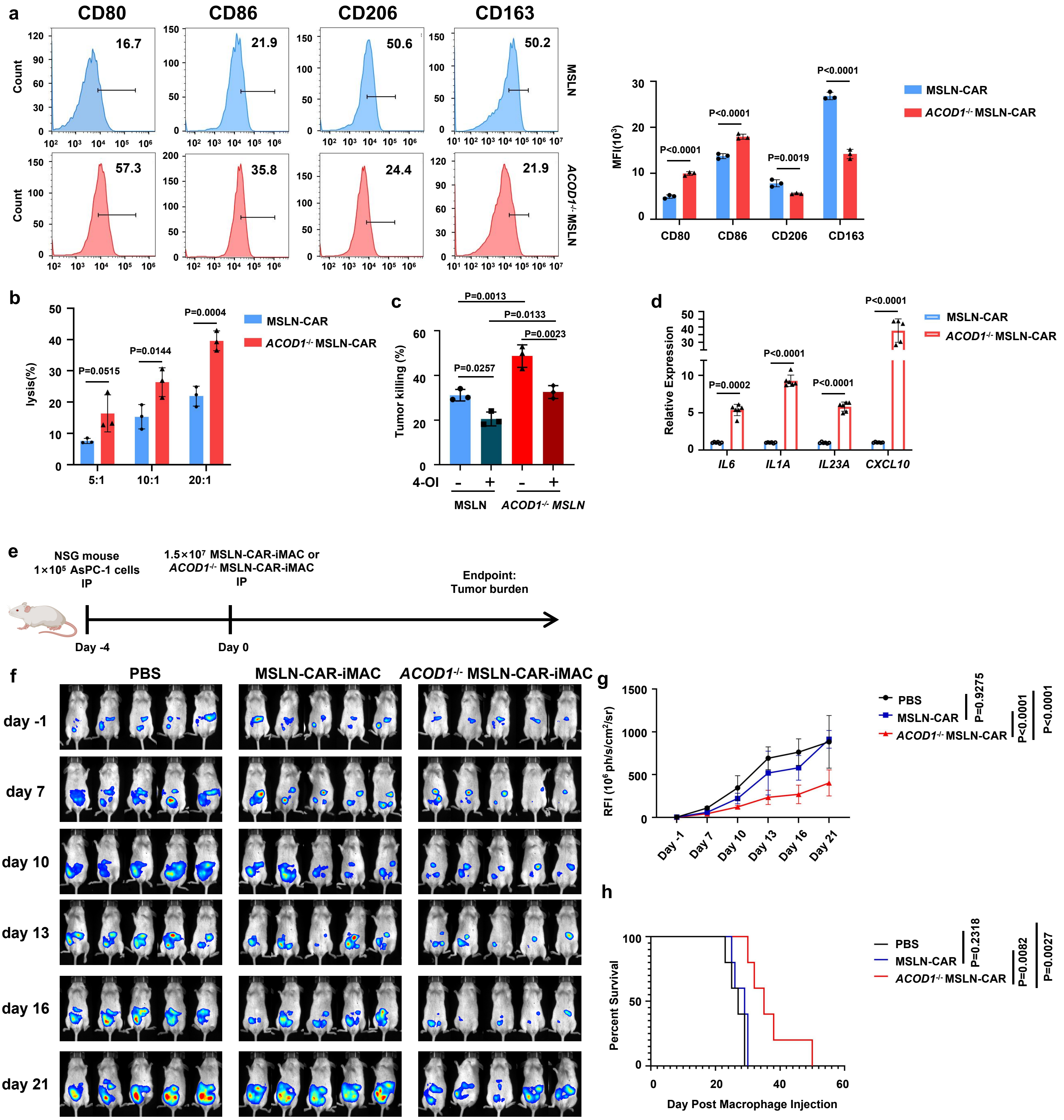

Supplementary Fig. 12 | *ACOD1* deletion promoted anti-pancreatic cancer activity of iMACs in vitro and in vivo, related to **Fig. 6**. **a**, The expression of CD80, CD86, CD206, and CD163 in MSLN-CAR-iMACs or *ACOD1*$^{-/-}$ MSLN-CAR-iMACs after co-cultured with pancreatic cancer cell AsPC-1 (E:T=5:1) for 24 h measured by flow cytometry and the representative data was displayed as histograms (left). Data was shown as mean ± SD, n=3 biologically independent samples. Statistics by two-way ANOVA test (CD80, P<0.0001; CD86, P<0.0001; CD206, P=0.0019; CD163, P<0.0001). **b**, Luciferase assays for CAR-iMAC cytotoxicity activity against cancer cells when co-cultured with AsPC-1 cells for 24 h (E:T=5:1, 10:1, or 20:1) (Data was shown as mean ± SD, n=3 biologically independent samples). Statistics by two-way ANOVA test (5:1, P=0.0515; 10:1, P=0.0144; 20:1, P=0.0004). **c**, Luciferase assays for CAR-iMAC cytotoxicity activity against cancer cells with or without 4-Octyl Itaconate (4-OI) (250 μM) when co-cultured with AsPC-1 cells for 24 h (E: T=10:1) (Data was shown as mean ± SD, n=3 biologically independent samples). Statistics by one-way ANOVA test. **d**, qRT-PCR for mRNA expression of pro-inflammatory genes in MSLN-CAR-iMACs or *ACOD1*$^{-/-}$ MSLN-CAR-iMACs after co-cultured with AsPC-1 cells (E: T=5:1) for 24 h (Data was shown as mean ± SD, n=3 biologically independent samples). Statistics by two-way ANOVA test (*IL6*, P=0.0002; *IL1A*, P<0.0001; *IL23A*, P<0.0001; *CXCL10*, P<0.0001). **e**, A diagram of the *in vivo* treatment scheme. **f**, In Vivo Imaging system (IVIS) images showing progression of tumor (n=5 per group). **g**, Tumor burden on day -1, 7, 10, 13, 16 and 21 was quantified and displayed as mean ± SD. Statistics by two-way ANOVA test (PBS vs MSLN-CAR, P=0.9275; PBS vs *ACOD1*$^{-/-}$ MSLN-CAR, P<0.0001; MSLN-CAR vs *ACOD1*$^{-/-}$ MSLN-CAR, P<0.0001). **h**, The Kaplan-Meier curve demonstrating survival of the mice. Statistics by two-tailed log-rank test. Source data are provided as a Source Data file.

Supplementary Figure 13

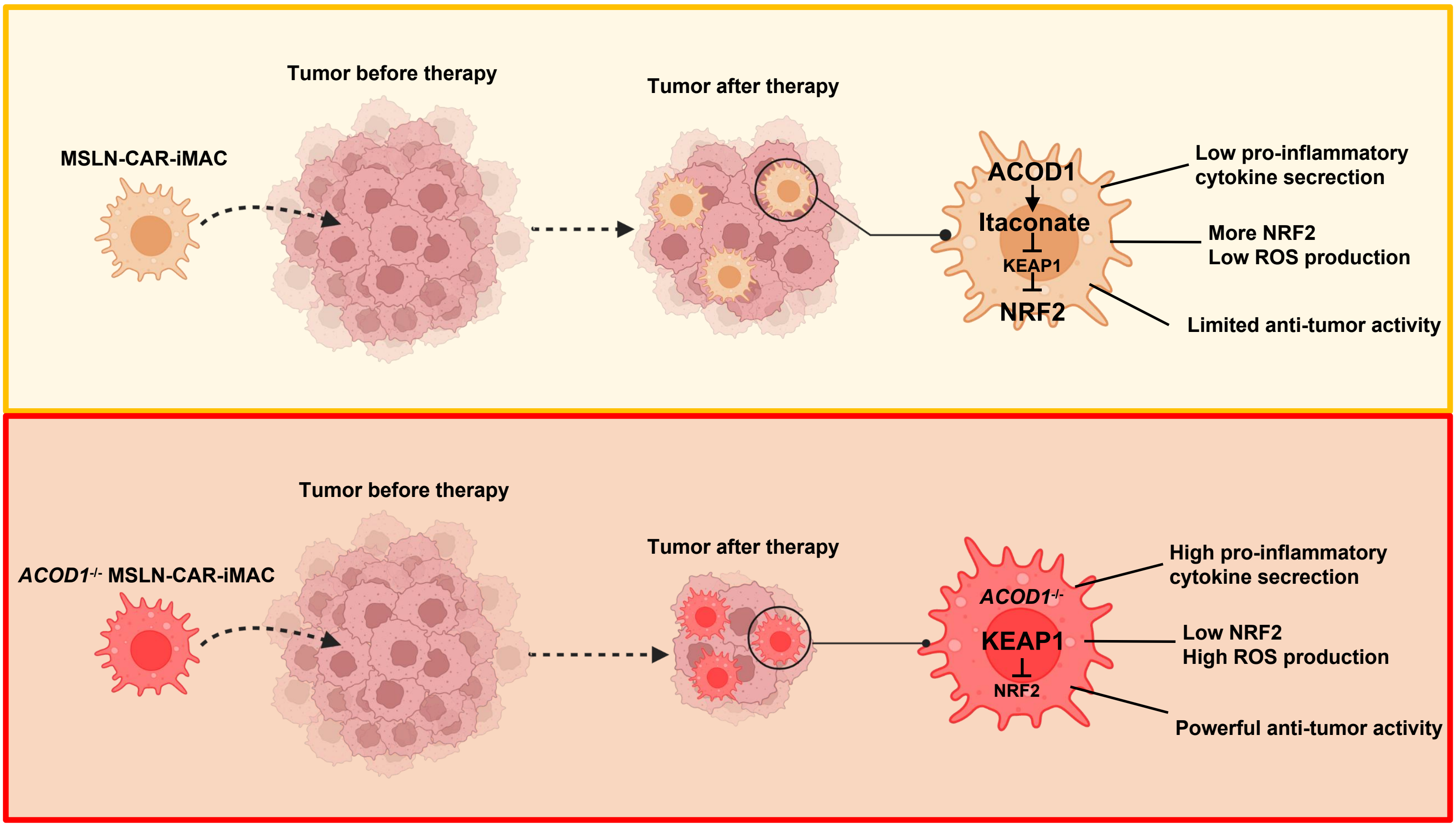

Supplementary Fig. 13 | The diagram of ACOD1 regulating the anti-tumor effect of MSLN-CAR-iMACs. MSLN-CAR-iMACs and *ACOD1*$^{-/-}$ MSLN-CAR-iMACs were activated upon stimulation with LPS and IFN-γ. The expression of itaconate was abrogated by *ACOD1* deletion in *ACOD1*$^{-/-}$ MSLN-CAR-iMACs. Itaconate is known to alkylate cysteine residues on KEAP1, promoting the accumulation and nuclear translocation of NRF2, which leads to the expression of downstream genes with anti-oxidant and anti-inflammatory properties. Consequently, *ACOD1*$^{-/-}$ MSLN-CAR-iMACs showed lower expression of NRF2, but higher levels of pro-inflammatory cytokines and reactive oxygen species (ROS). Furthermore, these cells exhibited enhanced M1-like polarization and stronger anti-tumor activity.

Supplementary Table 1. The sgRNAs used in this study.

name	5' to 3'	3' to 5'
ACOD1-1	CACCGCTGAATAACACGATCTGTC	AAACGACAGATCGTGTTATTCAGC
ACOD1-2	CACCGCACTTCCGTAGTGGTTCCC	AAACGGGAACCACTACGGAAGTGC
ACOD1-3	CACCGCGATTACTGCATTTCGCCA	AAACTGGCGAAATGCAGTAATCGC
ACOD1-4	CACCGTTACTGCATTTCGCCAAGG	AAACCCTTGGCGAAATGCAGTAAC
KEAP1-1	CACCGCCCACCCCGATGCGGTTACG	AAACCGTAACCGCATCGGGGTGGGC
KEAP1-2	CACCGCGTGCCCCGTAACCGCATCG	AAACCGATGCGGTTACGGGGCACGC
KEAP1-3	CACCGCGCCCGCGGTGTAGATCAGG	AAACCCTGATCTACACCGCGGGCGC
NRF2-1	CACCGGCATCTGATTTGGGAATGT	AAACACATTCCCAAATCAGATGCC
NRF2-2	CACCGACTGGGCTCTCGATGTGAC	AAACGTCACATCGAGAGCCCAGTC
NRF2-3	CACCGATACCGTCTAAATCAACAG	AAACCTGTTGATTTAGACGGTATC

Supplementary Table 2. The primers for qRT-PCR used in this study.

Gene	sequence
ACTIN-F	CATGTACGTTGCTATCCAGGC
ACTIN-R	CTCCTTAATGTCACGCACGAT
IL23A-F	CTCAGGGACAACAGTCAGTTC
IL23A-R	ACAGGGCTATCAGGGAGCA
IL6-F	ACTCACCTCTTCAGAACGAATTG
IL6-R	CCATCTTTGGAAGGTTCAGGTTG
CCR5-F	TTCTGGGCTCCCTACAACATT
CCR5-R	TTGGTCCAACCTGTTAGAGCTA
CCR7-F	TGAGGTCACGGACGATTACAT
CCR7-R	GTAGGCCCACGAAACAAATGAT
IL1A-F	AGATGCCTGAGATACCCAAAACC
IL1A-R	CCAAGCACACCCAGTAGTCT
IL1B-F	TCAGCCAATCTTCATTGCTCAA
IL1B-R	TGGCGAGCTCAGGTACTTCTG
CXCL9-F	CCAGTAGTGAGAAAGGGTCGC
CXCL9-R	AGGGCTTGGGGCAAATTGTT
CXCL10-F	TTCCTGCAAGCCAATTTTGTC
CXCL10-R	TCTTCTCACCCTTCTTTTTCATTGT
CXCL11-F	GACGCTGTCTTTGCATAGGC
CXCL11-R	GGATTTAGGCATCGTTGTCCTTT
TNF-F	GCAGGTCTACTTTGGGATCATTG
TNF-R	GCGTTTGGGAAGGTTGGA
NRF2-F	TTCCCGGTCACATCGAGAG
NRF2-R	TCCTGTTGCATACCGTCTAAATC
SOD2-F	GTTGGGGTTGGCTTGGTTTC
SOD2-R	GCCTGTTGTTCCTTGCAGTG
HMOX1-F	AAGACTGCGTTCCTGCTCAAC
HMOX1-R	AAAGCCCTACAGCAACTGTCG
GCLM-F	CATTTACAGCCTTACTGGGAGG
GCLM-R	ATGCAGTCAAATCTGGTGGCA
NQO1-F	GAAGAGCACTGATCGTACTGGC
NQO1-R	GGATACTGAAAGTTCGCAGGG
GSR-F	CACTTGCGTGAATGTTGGATG
GSR-R	TGGGATCACTCGTGAAGGCT
