# Supplementary material for: Metabolic Reprogramming via ACOD1 depletion enhances function of human induced pluripotent stem cell-derived CAR-macrophages in solid tumors
Source: Nat Commun. 2023 Sep 18;14:5778. doi: 10.1038/s41467-023-41470-9 (PMC10507032; doi:10.1038/s41467-023-41470-9)
Supplement: Supplementary file 3 — Reporting Summary [file 41467_2023_41470_MOESM3_ESM.pdf]

nature portfolio

Corresponding author(s): JIN ZHANG

Last updated by author(s): Aug 31, 2023

Reporting Summary

Nature Portfolio wishes to improve the reproducibility of the work that we publish. This form provides structure for consistency and transparency in reporting. For further information on Nature Portfolio policies, see our Editorial Policies and the Editorial Policy Checklist.

Statistics

For all statistical analyses, confirm that the following items are present in the figure legend, table legend, main text, or Methods section.

n/a	Confirmed	
☐	☒	The exact sample size (*n*) for each experimental group/condition, given as a discrete number and unit of measurement
☐	☒	A statement on whether measurements were taken from distinct samples or whether the same sample was measured repeatedly
☐	☒	The statistical test(s) used AND whether they are one- or two-sided<br>*Only common tests should be described solely by name; describe more complex techniques in the Methods section.*
☒	☐	A description of all covariates tested
☐	☒	A description of any assumptions or corrections, such as tests of normality and adjustment for multiple comparisons
☐	☒	A full description of the statistical parameters including central tendency (e.g. means) or other basic estimates (e.g. regression coefficient) AND variation (e.g. standard deviation) or associated estimates of uncertainty (e.g. confidence intervals)
☐	☒	For null hypothesis testing, the test statistic (e.g. *F*, *t*, *r*) with confidence intervals, effect sizes, degrees of freedom and *P* value noted<br>*Give P values as exact values whenever suitable.*
☒	☐	For Bayesian analysis, information on the choice of priors and Markov chain Monte Carlo settings
☒	☐	For hierarchical and complex designs, identification of the appropriate level for tests and full reporting of outcomes
☐	☒	Estimates of effect sizes (e.g. Cohen's *d*, Pearson's *r*), indicating how they were calculated

Our web collection on statistics for biologists contains articles on many of the points above.

Software and code

Policy information about availability of computer code

Data collection	1. Sequencing: Illlumina Hiseq 2500<br>2. Immunofluorescence: Olympus FV3000 microscope<br>3. Western blot: ChemiDoc Touch Imaging System (Bio-Rad)<br>4. Mitochondrial function and respiration: Seahorse XFe96 Analyzer (Agilent)<br>5. Bioluminescence imaging: IVIS Imaging System (BiospaceLab photonimager)<br>6. Luciferase assay: microplate reader (TECAN, SPARK)
Data analysis	ImageJ-win64, GraphPad Prism 9.0.0, FlowJo V10, WAVE V2.6 and MAGeCK softwares

For manuscripts utilizing custom algorithms or software that are central to the research but not yet described in published literature, software must be made available to editors and reviewers. We strongly encourage code deposition in a community repository (e.g. GitHub). See the Nature Portfolio guidelines for submitting code & software for further information.

Data

Policy information about availability of data

All manuscripts must include a data availability statement. This statement should provide the following information, where applicable:

- Accession codes, unique identifiers, or web links for publicly available datasets
- A description of any restrictions on data availability
- For clinical datasets or third party data, please ensure that the statement adheres to our policy

The data used to generate the main results are shown in main Figs and Supplementary Figs are available as supplementary information. Source data include uncropped western blots. All data supporting the findings of this study are available in a publicly accessible repository. The RNA-seq data that support the findings of this study have been deposited in the Gene Expression Omnibus (GEO) under the following accession codes: GSE216352 (Web address is https://www.ncbi.nlm.nih.gov/geo/query/acc.cgi). The pooled screen data have been deposited in GEO under the accession number: GSE216353 (Web address is https://www.ncbi.nlm.nih.gov/geo/query/acc.cgi). Both are under the accession number: GSE216354 (Web address is https://www.ncbi.nlm.nih.gov/geo/query/acc.cgi?acc=GSE216354). Source data are provided with this paper.

Research involving human participants, their data, or biological material

Policy information about studies with human participants or human data. See also policy information about sex, gender (identity/presentation), and sexual orientation and race, ethnicity and racism.

Reporting on sex and gender	*Use the terms sex (biological attribute) and gender (shaped by social and cultural circumstances) carefully in order to avoid confusing both terms. Indicate if findings apply to only one sex or gender; describe whether sex and gender were considered in study design; whether sex and/or gender was determined based on self-reporting or assigned and methods used. Provide in the source data disaggregated sex and gender data, where this information has been collected, and if consent has been obtained for sharing of individual-level data; provide overall numbers in this Reporting Summary. Please state if this information has not been collected. Report sex- and gender-based analyses where performed, justify reasons for lack of sex- and gender-based analysis.*
Reporting on race, ethnicity, or other socially relevant groupings	*Please specify the socially constructed or socially relevant categorization variable(s) used in your manuscript and explain why they were used. Please note that such variables should not be used as proxies for other socially constructed/relevant variables (for example, race or ethnicity should not be used as a proxy for socioeconomic status). Provide clear definitions of the relevant terms used, how they were provided (by the participants/respondents, the researchers, or third parties), and the method(s) used to classify people into the different categories (e.g. self-report, census or administrative data, social media data, etc.) Please provide details about how you controlled for confounding variables in your analyses.*
Population characteristics	*Describe the covariate-relevant population characteristics of the human research participants (e.g. age, genotypic information, past and current diagnosis and treatment categories). If you filled out the behavioural & social sciences study design questions and have nothing to add here, write "See above."*
Recruitment	*Describe how participants were recruited. Outline any potential self-selection bias or other biases that may be present and how these are likely to impact results.*
Ethics oversight	*Identify the organization(s) that approved the study protocol.*

Note that full information on the approval of the study protocol must also be provided in the manuscript.

Field-specific reporting

Please select the one below that is the best fit for your research. If you are not sure, read the appropriate sections before making your selection.

☒ Life sciences ☐ Behavioural & social sciences ☐ Ecological, evolutionary & environmental sciences

For a reference copy of the document with all sections, see nature.com/documents/nr-reporting-summary-flat.pdf

Life sciences study design

All studies must disclose on these points even when the disclosure is negative.

Sample size	No sample size calculation was performed. Sample size was determined on experiment feasibility and material availability. At least three biological replicates are available to calculate the statistical significance.
Data exclusions	No data was excluded from the analysis.
Replication	All experimental findings were reproduced as biological replicates at the value stated in figure legends, unless otherwise indicated. All additional replication attempts were consistent between each other.
Randomization	Cell samples and research animals were randomly assigned to control and treatment groups.

Blinding	No specific blinding was applied since all experiments were assigned into groups including relevant controls and analysis was done objectively and without bias.

Reporting for specific materials, systems and methods

We require information from authors about some types of materials, experimental systems and methods used in many studies. Here, indicate whether each material, system or method listed is relevant to your study. If you are not sure if a list item applies to your research, read the appropriate section before selecting a response.

Materials & experimental systems

n/a	Involved in the study
☐	☒ Antibodies
☐	☒ Eukaryotic cell lines
☒	☐ Palaeontology and archaeology
☐	☒ Animals and other organisms
☒	☐ Clinical data
☒	☐ Dual use research of concern
☒	☐ Plants

Methods

n/a	Involved in the study
☒	☐ ChIP-seq
☐	☒ Flow cytometry
☒	☐ MRI-based neuroimaging

Antibodies

Antibodies used	APC anti-human CD206 (Biolegend, Cat: 321109, Clone: 15-2, Lot: B348965), APC anti-human CD86 (Biolegend, Cat: 305411, Clone:IT2.2, Lot: B351349), PE anti-human CD80 (Biolegend, Cat:305208, Clone: 2D10, Lot: B330518), PE anti-human CD163 (Biolegend, Cat:333606, Clone: GHI/61, Lot: B347256), FITC anti-human CD14 (Biolegend, Cat: 325604, Clone: HCD14, Lot: B268830) and APC anti-humanCD11B (Biolegend, Cat: 301309, Clone: ICRF44, Lot: B278346), PE Mouse IgG1, κ isotype (Biolegend, Cat: 400113, Clone: MOPC-21, Lot: B245984), APC Mouse IgG1, κ isotype (Biolegend, Cat: 400119, Clone: MOPC-21, Lot: B243042), FITC Mouse IgG1, κ isotype (Biolegend, Cat: 400107, Clone: MOPC-21, Lot: B199152), HRP AffiniPure Goat anti-Rabbit IgG (H+L) secondary antibody (EARTHOX, Cat: 620822), β-Actin (13E5) Rabbit mAb (Cell Signaling Technology, Cat: #4970), NRF2 (D1Z9C) Rabbit mAb (Cell Signaling Technology, Cat: #12721), Anti-Keap1 antibody (abcam, Cat: ab227828), Anti-IRG1 antibody (abcam, Cat: ab222411), InVivoMAb human IgG1 isotype control (BioXcell, BE0297), InVivoMAb anti-human IFNγ (BioXcell, BE0235), InVivoSIM anti-human TNFα (BioXcell, SIM0006), Anti-PD1 antibody (Sintilimab)(Chemstan, Cat: CSD00572) and anti-CD47 antibody was a gift from Biocytogen Pharmaceuticals (Beijing).
Validation	The antibodies used in this study are commercially available and were used for the applications validated by manufactures.<br>β-Actin (13E5) Rabbit mAb: Applications: WB,IHC-P, IF-IC, F. Reactivity: H, M, R, Mk, B,Pg. This antibody was validated as their website stating "Western blot analysis of cell extracts from various cell lines using beta-Actin (13E5) Rabbit mAb".<br>NRF2 (D1Z9C) Rabbit mAb: Applications: WB,IP, IF-IC, F, ChIP, ChIP-seq. Reactivity: H, M, Mk. This antibody was validated as their website stating "Western blot analysis of extracts from MEF wt and U-2 OS cells, untreated (-) or treated with MG-132 #2194 (10 µM, 10 hr; +)".<br>Anti-Keap1 antibody: Applications: WB,IHC-P. Reactivity: H, M. This antibody was validated by Knock-out (KO) validation.<br>Anti-IRG1 antibody : Applications: WB,IP. Reactivity: H, M. This antibody was validated by RAW264.7 and THP-1 cells treated or untreated with lipopolysaccharide.

Eukaryotic cell lines

Policy information about cell lines and Sex and Gender in Research

Cell line source(s)	HEK293T (ATCC CRL-3216), THP-1 (ATCC TIB-202), K562 (ATCC CCL-243), Nalm6 (ATCC CRL-3273) and AsPC-1 (ATCC CRL-1682) were obtained from the National Collection of Authenticated Cell Cultures and cultured according to standard protocols. HO-8910 (MZ-0089) was purchased from NingboMingZhoubioCO.,Ltd (Zhejiang, China). Human iPSCs were obtained from the reprogramming of peripheral blood mononuclear cells from a volunteer donor. The experiment was approved by the Human Subjects Committee of Jinling Hospital, Nanjing University. Approval number: 2020DZSKTZX-007.
Authentication	Human iPSC have been authenticated by morphology, immunofluorescence, flow cytometry and teratoma formation assay.
Mycoplasma contamination	All cell lines were tested negative for mycoplasma contamination.
Commonly misidentified lines (See ICLAC register)	No commonly misidentified cell lines were used.

Animals and other research organisms

Policy information about studies involving animals; ARRIVE guidelines recommended for reporting animal research, and Sex and Gender in Research

Laboratory animals	6–8-week-old NOD/SCID/IL2rγnull (NSG) mice (Gempharmatech, Jiangsu) were maintained under pathogen-free

Laboratory animals	conditions under the Zhejiang University Institutional Animal Care and followed the committee's approved protocols. All mice were maintained in suitable temperature (25℃) and humidity environment (typically 50%), 12 hour dark/light cycle, and fed with sufficient water and food.
Wild animals	No wild animals were used in this study.
Reporting on sex	Female mice were used in ovarian cancer model and male mice were used in pancreatic cancer model.
Field-collected samples	No field collected samples were used in this study.
Ethics oversight	All the mice were maintained under a specific pathogen-free (SPF) conditions. All experimental procedures were performed in accordance with the Animal Research Committee guidelines of Zhejiang University.

Note that full information on the approval of the study protocol must also be provided in the manuscript.

Flow Cytometry

Plots

Confirm that:

☒ The axis labels state the marker and fluorochrome used (e.g. CD4-FITC).

☒ The axis scales are clearly visible. Include numbers along axes only for bottom left plot of group (a 'group' is an analysis of identical markers).

☒ All plots are contour plots with outliers or pseudocolor plots.

☒ A numerical value for number of cells or percentage (with statistics) is provided.

Methodology

Sample preparation	Cells were re-suspended in FACS buffer (PBS with 2.5% FBS) and incubated with Fc blocker for 10 min on ice. After washing with FACS buffer, anti-CD80, anti-CD206, anti-CD86, anti-CD163 or anti-CD11B antibody (Biolegend, 1:100) were used for staining.
Instrument	DxFLEX flow cytometer (Beckman Coulter)
Software	Data were acquired on DxFLEX flow cytometer and analyzed with the FlowJo V10 software.
Cell population abundance	According to FSC/SSC and DAPI staining, the live cells can be gated. Cells were then gated on FSC-A vs SSC-A to discriminate doublets. The total analyzed cells accounts for more than 80% of total cells.
Gating strategy	Cells were gated on FSC-A/SSC-A. Cell debris and dead cells were removed from the analysis based on size and complexity. Cells were gated on FSC-A vs SSC-A to discriminate doublets. IgG was used as a negative control. For cells incubated with other antibodies, expression levels were represented by the fluorescence intensity.

☒ Tick this box to confirm that a figure exemplifying the gating strategy is provided in the Supplementary Information.
