# Supplementary material for: Metabolic Reprogramming via ACOD1 depletion enhances function of human induced pluripotent stem cell-derived CAR-macrophages in solid tumors
Source: Nat Commun. 2023 Sep 18;14:5778. doi: 10.1038/s41467-023-41470-9 (PMC10507032; doi:10.1038/s41467-023-41470-9)
Supplement: Supplementary file 2 — Peer Review File [file 41467_2023_41470_MOESM2_ESM.pdf]

nature portfolio

Peer Review File

Metabolic Reprogramming via ACOD1 depletion enhances function of human induced pluripotent stem cell-derived CAR-macrophages in solid tumors

Open Access This file is licensed under a Creative Commons Attribution 4.0 International License, which permits use, sharing, adaptation, distribution and reproduction in any medium or format, as long as you give appropriate credit to the original author(s) and the source, provide a link to the Creative Commons license, and indicate if changes were made. In the cases where the authors are anonymous, such as is the case for the reports of anonymous peer reviewers, author attribution should be to 'Anonymous Referee' followed by a clear attribution to the source work. The images or other third party material in this file are included in the article's Creative Commons license, unless indicated otherwise in a credit line to the material. If material is not included in the article's Creative Commons license and your intended use is not permitted by statutory regulation or exceeds the permitted use, you will need to obtain permission directly from the copyright holder. To view a copy of this license, visit http://creativecommons.org/licenses/by/4.0/.

Editorial Note: This manuscript has been previously reviewed at another journal that is not operating a transparent peer review scheme. This document only contains reviewer comments and rebuttal letters for versions considered at *Nature Communications*.

REVIEWERS' COMMENTS

Reviewer #1 (Remarks to the Author):

The authors have addressed my concerns.

Reviewer #3 (Remarks to the Author):

The revised work by Wang et. al has adequately addressed my questions and I have no further concerns.

Reviewer #4 (Remarks to the Author):

In this article, Wang and colleagues used metabolic reprogramming of induced pluripotent macrophages by ACOD1 knockout to enhance human mesothelin CAR-iMAC activity against two solid tumour models. Furthermore, they have demonstrated that combination with CD47 and PD1/PD-1 axis further enhances this activity. I believe that the previous reviewer comments have been adequately addressed to warrant publication of this results.
